# Scalable Fabrication of High‐Efficiency Thin‐Film Perovskite Solar Cells in Air via Polymer‐Mediated Synthesis of α‐FAPbI_3_ Microcrystals

**DOI:** 10.1002/adma.202522508

**Published:** 2026-02-05

**Authors:** Fan Shen, Chenxu Zhao, Jia Xu, Xunhui Wang, Jiale Chen, Huijing Liu, Pengchen Zou, Xuewei Liu, Yao Fu, Huifang Han, Kun Lang, Yijun Wang, Xingyu Gao, Zhaofu Fei, Hong Zhang, Paul J. Dyson, Jianxi Yao

**Affiliations:** ^1^ New Energy Generation National Engineering Research Center North China Electric Power University Beijing China; ^2^ Beijing Key Laboratory of Energy Safety and Clean Utilization North China Electric Power University Beijing China; ^3^ Institute of Chemical Sciences and Engineering École Polytechnique Fédérale de Lausanne (EPFL) Lausanne Switzerland; ^4^ Shanghai Synchrotron Radiation Facility (SSRF) Shanghai Advanced Research Institute Chinese Academy of Sciences Shanghai China; ^5^ State Key Laboratory of Photovoltaic Science and Technology Shanghai Frontiers Science Research Base of Intelligent Optoelectronics and Perception Institute of Optoelectronics College of Future Information Technology Fudan University Shanghai China

**Keywords:** additive engineering, in situ grazing incidence wide‐angle X‐ray scattering (GIWAXS), inverse temperature crystallization(ITC), perovskite solar cells(PSCs), scalable fabrication

## Abstract

Formamidinium lead triiodide (FAPbI_3_) perovskite solar cells (PSCs) demonstrate exceptional photovoltaic performance but face critical stability challenges impeding commercialization. Herein, we integrate polypropylene glycol (PPG) into an inverse temperature crystallization process to synthesize highly stable α‐FAPbI_3_ microcrystals, which retain phase purity for over six months in air. Our approach enables large‐scale production (nearly 50 g) of PPG‐coated α‐FAPbI_3_ (target) microcrystals from low‐cost PbI_2_, with over 95% yield—sufficient to manufacture 23 m^2^ of perovskite solar modules. Redissolving the target α‐FAPbI_3_ microcrystals, followed by spin‐coating and annealing, yields target perovskite films with reduced defect density, minimized residual strain, and enhanced carrier transport. Mechanistic investigations reveal that colloidal species form upon the redissolution of target microcrystals which modulate the film crystallization kinetics, i.e. accelerating (100)‐oriented nucleation and growth. Employing this integrated strategy, a champion PSC with a power conversion efficiency (PCE) of 26.50% (certified 26.22%) was obtained at a laboratory scale (0.06 cm^2^) and 22.66% for a module with an aperture area of 28.99 cm^2^, together with prolonged operational stability. This work opens new avenues for the industrial‐scale fabrication of efficient and stable large‐area perovskite photovoltaics.

## Graphical abstract

We report a scalable strategy using PPG‐coated α‐FAPbI_3_ microcrystals that may be processed in air and undergo controlled redissolution to form highly oriented, defect‐suppressed perovskite films. The PSCs derived from these films deliver a PCE of 26.5% and exhibit enhanced stability, demonstrating potential for industrial large‐scale perovskite photovoltaic manufacturing.

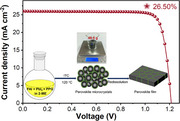

## 1 Introduction

Perovskite solar cells (PSCs) have emerged as a transformative photovoltaic technology, driven by unprecedented advancements in power conversion efficiency (PCE) – ascending from below 3.8% [1] to over 27.0% within a decade [2]. While diverse strategies have been developed to enhance performance, the fundamental origins of precursor stability remain inadequately understood, and precise control over individual stages of the perovskite film formation process is often lacking [3, 4, 5]. Consequently, long‐term device stability continues to represent a critical bottleneck hindering the commercialization of PSCs [6]. Among perovskite compositions, formamidinium lead triiodide (FAPbI_3_) stands out for its suitable bandgap and efficient charge transport, offering strong potential for high‐performance PSCs [7]. A particularly promising advancement for FAPbI_3_‐based devices is the precursor pre‐synthesis route, often termed the “crystal redissolution strategy” [8]. This method involves dissolving pre‐synthesized perovskite microcrystals to yield precursor solutions that are rich in well‐defined colloidal species, such as perovskite‐like clusters or pre‐assembled 3D inorganic scaffolds with organic cation insertion [9]. These pre‐nucleation entities facilitate a more direct and controlled phase transformation during subsequent film crystallization, usually leading to enhanced film quality [10, 11].

For the redissolution strategy to yield optimal films, the quality of the perovskite microcrystals prior to the redissolution step is crucial [12]. Inverse temperature crystallization (ITC) is highly effective for the preparation of high‐purity, single‐crystalline perovskite materials [13]. However, challenges persist with conventional α‐FAPbI_3_ microcrystals, which are prone to degradation due to intrinsic phase instability and sensitivity to environmental factors such as moisture, ultraviolet light, oxygen, and temperature, impeding the application of the crystal redissolution strategy [14, 15]. In practical industrial manufacture, long‐term stable α‐FAPbI_3_ microcrystals are required, especially during storage and transport. In addition, ultra‐pure PbI_2_ is extremely expensive and increases production costs [16, 17]. Recently, additive engineering has emerged as a key approach for stabilizing α‐FAPbI_3_, particularly with judiciously selected additives such as ammonium salts [18], ionic liquids [4], or polymers [19], which can effectively regulate crystal growth and control film formation. Notably, oxygen‐rich (O‐rich) polymers serve as functional additives for the synthesis of high‐quality α‐FAPbI_3_, ultimately delivering greatly improved stabilities. Rubidium‐functionalized polyacrylic acid has also been reported to regulate the crystallization of α‐FAPbI_3_, passivate defects, and suppress Pb^2+^ ion leakage through coordination with the carboxylic groups [20]. It has been shown that polypropylene glycol (PPG) can control nucleation through coordination of the oxygen atoms on the polymer with Pb^2+^ ions, resulting in large‐sized, high‐quality perovskite single crystals [21]. However, the use of PPG‐mediated α‐FAPbI_3_ microcrystals as raw materials for fabricating PSCs remains unexplored, and a scalable synthesis of microcrystals has not been reported. Despite multiple techniques being available to improve the quality of perovskite films, and hence boost the performance of the PSCs, precise control of the individual techniques in an integrated process that provides α‐FAPbI_3_ microcrystals with long‐term stability would be advantageous.

In this work, we combine additive engineering with ITC to prepare low‐cost, stable PPG‐coated α‐FAPbI_3_ (target) microcrystals on a large‐scale (48.50 g, sufficient to produce 23 m^2^ perovskite solar modules) [22, 23, 24]. Subsequent redissolution of the target microcrystals followed by spin‐coating and annealing, affords high‐quality perovskite films. The colloidal species formed upon redissolution of the target microcrystals accelerate (100)‐oriented nucleation and growth, producing films with enhanced crystallinity and reduced defect density. The resulting PSCs achieved a champion PCE of 26.50% (certified 26.22%) with exceptional long‐term operational stability, retaining over 92.6% of the initial PCE after 2880 hours without encapsulation under 1% relative humidity at 25°C in the dark. A module with an effective area of 28.99 cm^2^ and an efficiency of 22.66% was obtained using this technique.

## 2 Results and Discussion

### 2.1 Synthesis and Characterization of Stable Target α‐FAPbI_3_ Microcrystals

**FIGURE 1:**
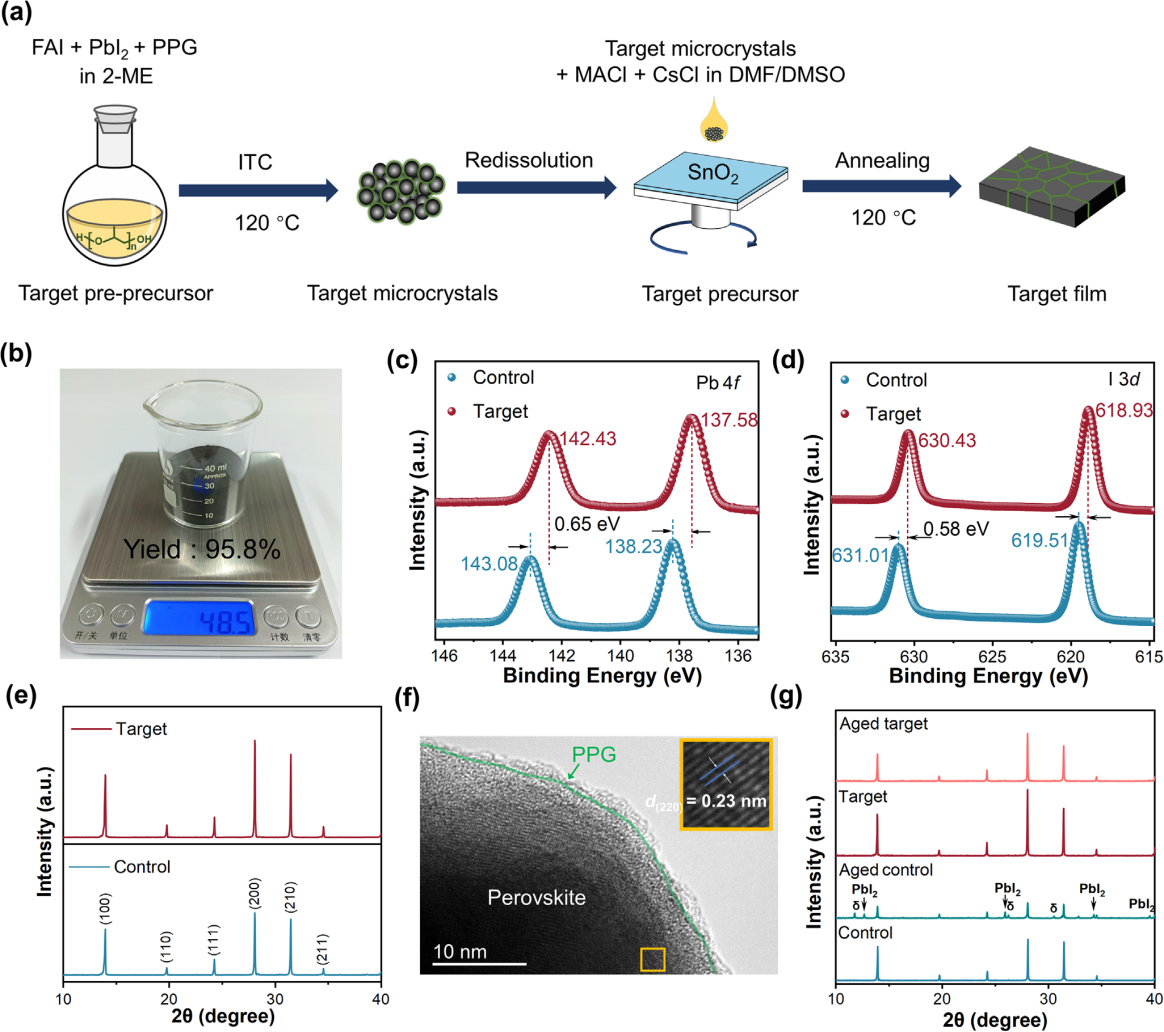
(a) Schematic of the preparation of target microcrystals using the ITC method and the preparation of target perovskite films. (b) Photograph of the ca. 48.50 g scale synthesized target α‐FAPbI_3_ microcrystals. (c) Pb 4*f* and (d) I 3*d* XPS spectra of the target and control microcrystals. (e) XRD patterns of the target and control microcrystals. (f) HRTEM image of target microcrystals (interplanar distance *d*_(220)_ = 0.23 nm). (g) XRD patterns of freshly prepared and 6‐month aged microcrystals.

Employing the ITC method, a target pre‐precursor solution consisting of lead iodide (99% PbI_2_), formamidinium iodide (FAI), and PPG (1.0 mol% PPG relative to PbI_2_) dissolved in 2‐methoxyethanol (2‐ME) was heated at 120 °C, leading to the formation of the target microcrystals, i.e., α‐FAPbI_3_ coated with PPG (Figure 1a). The resulting target microcrystals were redissolved in DMF/DMSO (7.5:1 in volume ratio) with 20 mol% MACl and 5 mol% CsCl additives (relative to PbI_2_), forming the target precursor solution. The target precursor solution was spin‐coated onto SnO_2_ films and annealed at 120°C, to afford a high‐quality perovskite (target) film (Figure 1a). This entire process was carried out in ambient air at 25°C and ∼30% relative humidity (RH). Microcrystals without PPG and the corresponding perovskite films were also prepared as controls. The PCE of PSCs is strongly dependent on the purity of the precursors, in particular, the purity of PbI_2_, with high‐performance PSCs with PCEs >26% prepared from PbI_2_ with purities >99.99% (Table S1). Although the synthesis of large‐scale δ‐FAPbI_3_ (ca. 1 kg) [25] have been reported, bulk production of α‐FAPbI_3_ microcrystals is elusive. For this reason, we upscaled the synthesis of the target α‐FAPbI_3_ microcrystals to the 48.50 g scale (Figure 1b) (see Note S1). Samples used in this study for PSCs fabrication were taken from this bulk production. Specific usage rates of α‐FAPbI_3_ microcrystals in PSCs are not routinely reported as the actual amount of α‐FAPbI_3_ microcrystals used depends on the specific fabrication method.

According to the x‐ray photoelectron spectroscopy (XPS) analysis [26], the Pb 4*f* characteristic peaks in the control microcrystals are observed at 138.23 and 143.08 eV, whereas the target microcrystals exhibit peaks at slightly lower binding energies of 137.58 and 142.43 eV (Figure 1c). Similarly, the I 3*d* peaks for target microcrystals are observed at lower binding energies relative to those in control, i.e. shifting from 619.51 and 631.01 to 618.93 and 630.43 eV (Figure 1d). The O 1*s* spectrum contains a weak signal at 532.74 eV for control microcrystals, attributable to surface‐adsorbed oxygen species [27], whereas the target microcrystals exhibit a pronounced peak at 533.24 eV originating from the oxygen atoms in PPG (Figure S1). The shift of the Pb 4*f* and I 3*d* peaks to lower binding energies, together with the distinct O 1*s* response, strongly supports the presence of interactions between PPG and Pb^2+^ [21, 28]. The shift in binding energy of I 3*d* could be a result of Pb‐O interactions which influence the coordination sphere of the Pb^2+^ center. X‐ray Diffraction (XRD) patterns of the control and target microcrystals contain characteristic peaks of α‐FAPbI_3_ at identical positions (Figure 1e) [21, 29], indicating that the PPG does not disrupt the α‐FAPbI_3_ crystal structure. This is not unexpected as the polymer is not expected to penetrate the FAPbI_3_ lattice [30]. Thus, PPG accumulates at the surface or in the boundaries of the FAPbI_3_ perovskite crystals (Figure S2). However, the peaks in the target microcrystals are more intense than those of the control, suggesting that incorporation of PPG enhances the formation of the α‐FAPbI_3_ microcrystals (Figure 1e). It is noteworthy that the crystallinity of target microcrystals depends on the amount of the PPG used, with the optimal concentration being 1.0 mol% PPG relative to the PbI_2_ (Figure S3). Characterization of the target microcrystals and subsequent target films and devices are all based on this optimal concentration. Furthermore, the target microcrystals were prepared at 25°C under high‐humidity conditions (∼60% RH), and their phase purity was examined by XRD. The characteristic diffraction peaks of the α‐FAPbI_3_ phase are well preserved and the emergence of secondary phases are not observed (Figure S4), demonstrating the high tolerance of the microcrystal formation process to humidity.

High‐resolution transmission electron microscopy (HRTEM) confirms that the target microcrystals maintain a cubic α‐phase perovskite structure, characterized by α‐FAPbI_3_ grains with an interplanar distance of *d*_(220)_ at 0.23 nm (Figure 1f), which is within the range of reported values [31, 32]. Thermogravimetric (TG) and derivative thermogravimetric (DTG) analyses reveal two weight‐loss stages for both the control and target microcrystals (Figure S5). The first mass loss, assigned to FAI decomposition, occurs at 333°C for the control microcrystals and increases to 342°C for the target microcrystals [33]. The second mass loss, corresponding to the breakdown of the perovskite lattice and PbI_2_ volatilization, is observed at 567°C and 595°C, respectively.

To evaluate phase stability, the control and target microcrystals were stored under ambient air (25°C, 40 ∼ 50% RH) for 6 months. XRD analysis revealed that the aged target microcrystals retained the α‐FAPbI_3_ phase, whereas aged control microcrystals showed clear signs of degradation, forming the δ‐FAPbI_3_ phase (i.e. new peaks emerged at 11.75°, 26.24° and 30.53°) and PbI_2_ (peaks at 12.65°, 25.91°, 34.26° and 39.50°) (Figure 1g). The higher stability of the target microcrystals compared to the control microcrystals was confirmed by XPS analysis. After 6 months of ambient storage, aged control microcrystals exhibit pronounced changes, including the appearance of peaks attributable to Pb^0^ (136.12 (4*f*_7/2_) and 141.02 eV (4*f*_5/2_)) [34] and a decrease in the I:Pb ratio from 2.78 to 1.87 (calculated from the integrated I 3*d* and Pb 4*f* peak areas normalized by relative sensitivity factors), indicating partial decomposition and iodide loss (Figure 1c,d; Figure S6). In contrast, the target microcrystals showed only minimal shifts in binding energies, with the I:Pb ratio slightly decreasing from 2.98 to 2.90 (Figure 1c,d; Figure S6).

### 2.2 Redissolution of the Perovskite Microcrystals and Perovskite Film Formation

The control and target microcrystals were redissolved in DMF/DMSO (7.5:1 v/v), to afford solutions exhibiting a typical Tyndall effect [35] indicative of the presence of colloidal particles (Figure S7). Dynamic light scattering (DLS) revealed that both the control and target precursor solutions contained colloidal particles comprising two size regimes. The smaller colloidal particles in both solutions are comparable in size (ca. 2 ± 0.76 nm) [36], whereas the larger colloidal particles in the target precursor solution (centered at 3491 nm) are significantly larger than those in the control (centered at 1053 nm) (Figure S8). The larger colloidal particles in solution influence the crystallization kinetics [37], promoting nucleation, and resulting in the formation of higher crystalline films with fewer defects [38].

**FIGURE 2:**
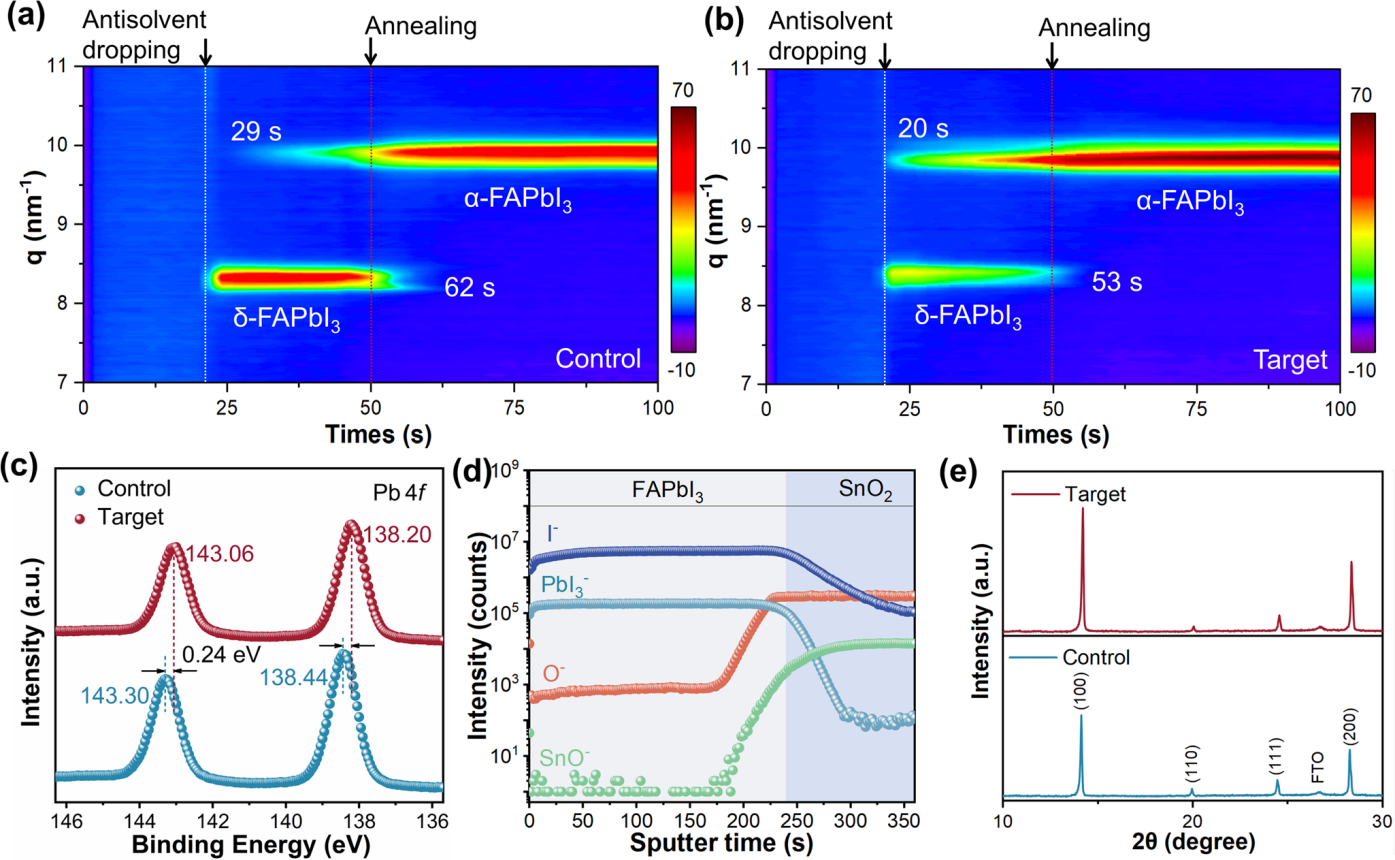
In situ GIWAXS spectra obtained during the crystallization of the (a) control and (b) target films. (c) XPS spectra of Pb 4*f* for the various perovskite films. (d) TOF‐SIMS depth profile of the target film deposited on FTO/SnO_2_ substrate. (e) XRD patterns of the control and target perovskite films.

Following spin‐coating of the precursor solutions onto SnO_2_ films, the crystallization process of perovskite films was monitored through in situ grazing incidence wide‐angle x‐ray scattering (GIWAXS) and in situ photoluminescence (PL) spectroscopy [39]. Antisolvent was applied to all films after 20 seconds during spin‐coating, with annealing after 50 seconds (starts at the 51st second). A diffraction peak at q = 8.26 nm^−1^, corresponding to δ‐FAPbI_3_, emerged in both the control (Figure 2a) and target (Figure 2b) films upon the addition of the antisolvent. Its formation is markedly suppressed in the target film. In the control film (Figure 2a), the diffraction peak at q = 10 nm^−1^, corresponding to the α‐FAPbI_3_ phase, did not appear until 29 seconds. In contrast, the target film (Figure 2b) exhibited an immediate and substantially stronger α‐FAPbI_3_ signal, showing accelerated nucleation and increased α‐FAPbI_3_ signal intensity in the target film (Figure S9).

Because the patterns are symmetrical, the data from 0 to 90° provide the complete orientation distribution whereas the data from 90 to 180° reflect the symmetric diffractions [40]. The control film displays two dominant orientations at azimuthal angles of 90 and 37° (143°)(Figure S10a), whereas the target film exhibits a dominant orientation at 90° and a weaker one at 43° (137°) (Figure S10b), indicating preferential crystallization along the α‐FAPbI_3_ (100) plane. The in situ GIWAXS analysis confirms that target microcrystals suppress δ‐FAPbI_3_ formation, accelerate α‐FAPbI_3_ nucleation, and induce preferential (100) orientation. The enhanced crystallinity and orientation are expected to improve charge transport in the target perovskite film [41].

For comparison, the crystallization process of perovskite films prepared using a single‐step procedure by directly mixing FAI, PbI_2_ and PPG in DMF/DMSO was investigated using in situ GIWAXS (Figure S11). In contrast to the microcrystal redissolution route, the single‐step approach leads to the formation of film with markedly different phase evolution behavior, with the δ‐FAPbI_3_ intermediate persisting longer and the formation of the α‐FAPbI_3_ phase being significantly delayed. These results demonstrate that directly adding PPG to the precursor solution does not reproduce the crystallization regulation and phase‐purity control afforded by the microcrystal‐based redissolution approach.

The evolution of the PL intensity in the perovskite films during the spin‐coating and annealing processes was also studied. During the spin‐coating process, the luminescence peak redshifts from 650 nm to approximately 804 nm, which may be attributed to α‐FAPbI_3_ crystal growth (Figure S12) [42]. Notably, the α‐FAPbI_3_ luminescence peak appears after 28 seconds in the control film (Figure S12a), whereas in the target film it emerges after only 20  seconds (Figure S12b), indicating accelerated formation of the α‐FAPbI_3_ phase. During the annealing process, a strong α‐FAPbI_3_ luminescence peak appears in the perovskite films, followed by a rapid decrease in intensity (Figure S12), likely due to solvent migrating from the film bulk to the surface, leading to dissolution of the perovskite surface [43]. Compared to the control film (Figure S12a), the interaction between PPG and α‐FAPbI_3_ in the target film (Figure S12b) promotes solvent evaporation, accelerating the dissolution process (as PPG preferentially interacts with the perovskite instead of the solvent). Further annealing promotes the crystallization and growth of the dissolved material within the perovskite film. The luminescence peak at 804 nm of the control film (Figure S12a) emerges at 119 seconds, whereas that of the target film (Figure S12b) appears earlier at 90 seconds with higher PL intensity. Moreover, the target film undergoes much faster nucleation (with a higher PL intensity) during both spin‐coating and annealing compared to the control film, in good agreement with the in situ GIWAXS findings.

XPS (Figures 2c; Figure S13) of the control film displays Pb 4*f* peaks at 138.44 and 143.30 eV, as well as I 3*d* peaks at 619.43 and 630.93 eV. For the target film, the Pb 4*f* peaks are observed at lower binding energies of 138.20 and 143.06 eV as are the I 3*d* peaks with values of 619.06 and 630.56 eV, indicative of interactions between PPG and the perovskite. The I:Pb ratio increases from 2.67 in the control film to 2.92 in the target film, evidencing that PPG effectively stabilizes iodide [44]. The time‐of‐flight secondary ion mass spectrometry (TOF‐SIMS) analysis (Figure 2d), reveals the expected uniform distribution of depth profiles of PbI_3_^−^ and I^−^ ions in the perovskite layer [45]. The O^−^ signal attributable to PPG is distributed throughout the perovskite film, and exhibits a sharp increase upon reaching the SnO_2_ layer due to its intrinsically high oxygen content.

The (100) and (200) diffraction peaks of the target film at 14.21° and 28.39° are more intense than the analogous peaks in the control film (Figure 2e), indicating that PPG improves the crystallinity of the perovskite film. Scanning electron microscopy (SEM) analysis reveals morphological improvements in the target film compared to the control film, characterized by enhanced grain compactness, a larger average grain size, and a narrower grain‐size distribution (Figure S14a,b). Such morphological features are recognized to reduce grain‐boundary‐assisted nonradiative recombination and facilitate carrier transport in perovskite films [46, 47, 48]. Grain size statistics further confirm an increase in average grain size (D_ave_) from 630 nm in the control film to 790 nm in the target film (Figure S14c,d). The surface morphology and roughness of the target film, as revealed by the atomic force microscopy (AFM), are also superior to those of the control film. The control film has a root‐mean‐square roughness of 33.6 nm (Figure S15a), whereas the value for the target film is lower, i.e. 24.3 nm (Figure S15b), with a smoother morphology likely able to enhance interfacial contact with the hole transport layer (HTL), as widely reported in previous studies [49, 50, 51, 52, 53]. Overall, these improvements in crystallinity, grain size, and surface smoothness indicate that the target film possesses a higher quality.

Grazing incidence x‐ray diffraction (GIXRD) was used to investigate residual strain in both the control and target films. For the control film, the diffraction peak at the tilt angle (*ψ*) shifted from 6.36° at *ψ* = 0° to 6.09° at *ψ* = 40° (Figure S16a), whereas the target film exhibits a smaller shift, from 6.36° at *ψ* = 0° to 6.28° at *ψ* = 40° (Figure S16b). Further evaluation through the linear fit relationship of 2θ‐sin^2^ (ψ) of the perovskite films reveals a slope value drop from 0.658 in the control to 0.208 in the target film (Figure S16c). The decreased slope of the linear fit indicates reduced internal strain in the target film, beneficial for enhancing the structural stability of the perovskite lattice, as strain relaxation in perovskite lattices suppresses defect formation and ion migration, thereby playing a critical role in improving long‐term operational stability [54, 55, 56].

Density functional theory (DFT) calculations were conducted to investigate the interactions between PPG (Figure S17 and Note S2) and the perovskite. α‐FAPbI_3_ has an adsorption energy with PPG of –2.085 eV, indicating that the integrity of α‐FAPbI_3_ is preserved in solution [38]. In addition, PPG reduces the surface energy of α‐FAPbI_3_ for the (100) (Figure S18a) and (111) (Figure S18b) crystal planes from 0.091 to 0.064 eV Å^−2^, and from 0.082 to 0.068 eV Å^−2^ respectively, corresponding to reductions of 30% and 17%. Notably, the target crystals exhibit a strong preference for growth along the (100) plane during film formation, which promotes ordered crystal growth.

To further highlight the advantages of PPG over other oxygen‐containing polymers, i.e. polyethylene glycol (PEG) (Figure S19a) and polyvinyl alcohol (PVA) (Figure S19b). DFT calculations were extended to structurally similar polymers using their monomers as models to delineate likely interactions with α‐FAPbI_3_. The adsorption energy between the monomers and α‐FAPbI_3_ was estimated, with the PPG monomer having a value of −2.085 eV, which is stronger than the PEG monomer (−0.192 eV) and PVA monomer (−0.782 eV). Among the polymers analyzed, PPG forms the strongest interaction with α‐FAPbI_3_, confirming its superior surface‐binding and ability to stabilize and passivate α‐FAPbI_3_ microcrystals.

The control and target perovskite films show similar absorption edges near 804 nm in their ultraviolet‐visible (UV–vis) spectra, yielding an optical bandgap of 1.54 eV (Figure S20). Steady‐state photoluminescence (PL), confocal PL mapping, and time‐resolved photoluminescence (TRPL) were employed to further characterize the control and target films deposited on glass substrates. Both films exhibit identical PL peak positions at 804 nm (Figure S21a). However, the PL peak in the target film has a higher intensity than that in the control film. Confocal PL mapping further confirms the superior quality of the target film, with more uniform and brighter PL emission (Figure S21b). According to the TRPL analysis, the average charge lifetime (*τ*_ave_) for the control film is 3672 ns and in the target film it is 5322 ns (Figure S22, Table S2), indicating a lower rate of non‐radiative recombination and enhanced open‐circuit voltage (*V*_OC_). According to the ultraviolet photoelectron spectroscopy (UPS) measurements (Figure S23a), compared with the control film [Fermi level (*E*_F_) = −4.25 eV, conduction band minimum (*E*_CBM_) = −4.02 eV, valence band maximum (*E*_VBM_) = −5.56 eV], the target film has an *E*_F_ of −4.00 eV that is closer to its *E*_CBM_ (−3.91 eV) (Figure S23b), suggesting a more favorable electronic structure for electron extraction. Together with the higher *E*_VBM_ (−5.45 eV) (Figure S23b), this alignment facilitates electron transfer to SnO_2_ and hole extraction to spiro‐OMeTAD, while suppressing interfacial recombination and improving device performance. Thus, PPG effectively modulates the interfacial energy levels and improves carrier transmission.

### 2.3 Device Performance and Stability

#### 2.3.1 Photovoltaic Performance of the PSCs

**FIGURE 3:**
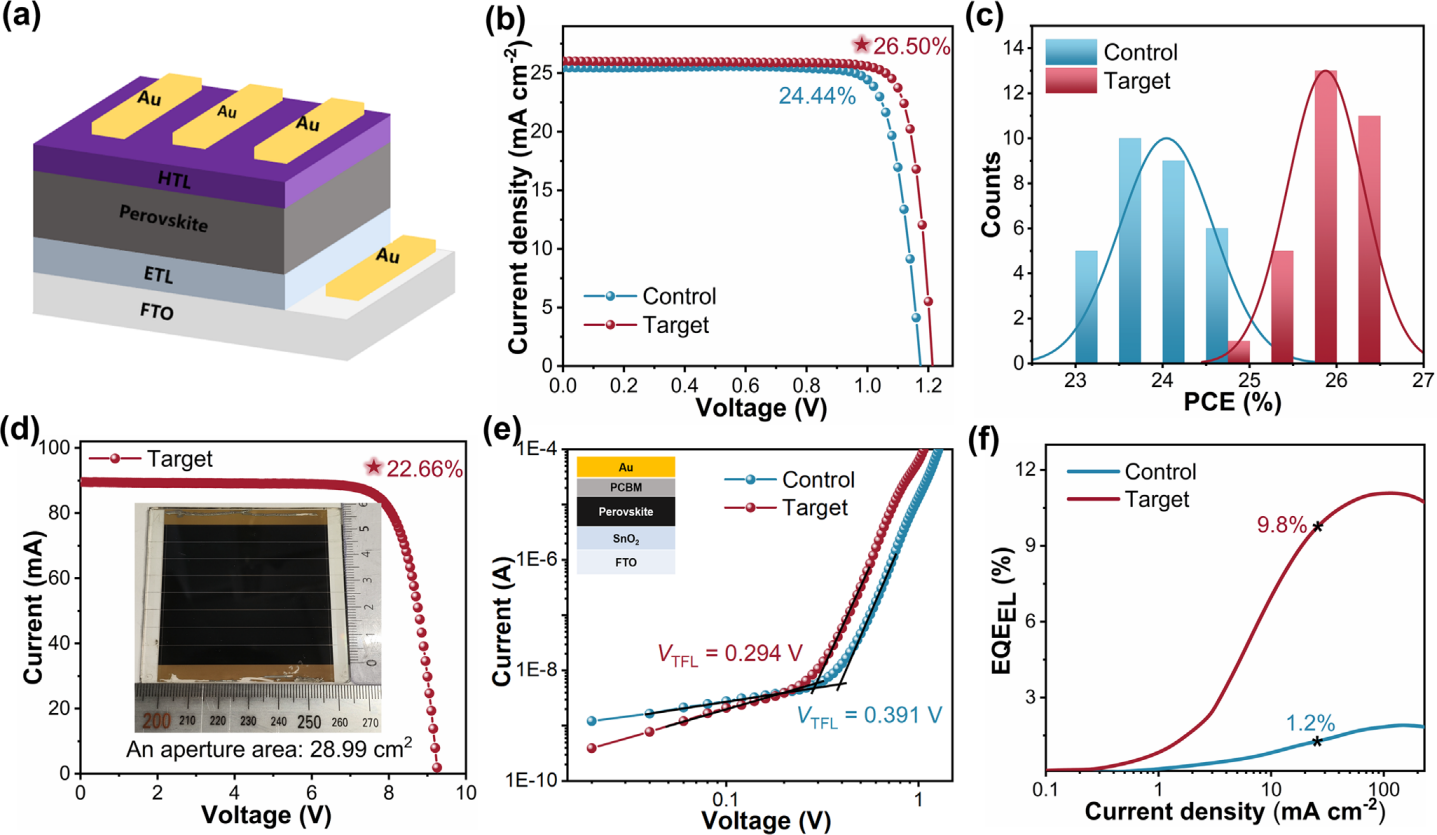
(a) Illustration of device structure. (b) *J–V* curves of the champion control and target devices under one‐sun illumination (100 mW/cm^2^). (c) Statistical distributions of PCEs from 30 control and 30 target devices. (d) *I–V* curve of the target perovskite solar module under reverse bias, the inset contains a photograph of the module. (e) SCLC curves of the electron‐only control and target devices. (f) EQE_EL_ of the control and target devices.

To evaluate the photovoltaic performance of the control and target PSCs, a regular device structure was applied, comprising FTO/SnO_2_/perovskite/spiro‐OMeTAD/Au (Figure 3a). The *J*−*V* characteristics under standard AM 1.5 G illumination for the champion control and target devices are shown in Figure 3b, with their respective photovoltaic parameters summarized in Table S3. The champion target device achieves a PCE of 26.50% (certified: 26.22%, Figure S24), considerably higher than that of the control device (24.44%). Notably, the open‐circuit voltage (*V*_OC_) increases from 1.174 V in the control device to 1.214 V in the target device (which is among the highest values reported for FAPbI_3_‐based devices [57]), along with improved short‐circuit current density (*J*_SC_) and fill factor (FF). The *J*−*V* plots of the champion target device measured in both forward and reverse scan directions are shown in Figure S25. PSCs were also fabricated entirely under high‐humidity conditions (∼60% RH), with the resulting devices achieving a high PCE of 25.41% (Figure S26 and Table S4), highlighting the excellent tolerance to humidity and industrial feasibility of the PPG‐coated α‐FAPbI_3_ microcrystals approach. To assess reproducibility, 30 control and 30 target devices were fabricated, and their PCEs are summarized in Figure 3c. The target devices display a narrow PCE distribution, with most exceeding 25%, highlighting the excellent reproducibility of the target PSCs. A stabilized PCE of 25.94% for the target device was maintained at maximum power point tracking (MPPT) for 300 seconds, exceeding that of the control device (22.61%, Figure S27). From external quantum efficiency (EQE) measurements, the integrated photocurrent densities for the champion control and target devices were calculated as 25.29 and 25.61 mA cm^−2^, respectively (Figure S28), closely matching the *J*_SC_ values from the *J*–*V* curves. Due to the uniformity and high‐quality crystallization required for large‐area perovskite films, we fabricated a perovskite solar module with an effective area of 28.99 cm^2^ and an efficiency of 22.66% (Figure 3d and Table S5), among the highest reported [58, 59]. Additionally, PVA‐ and PEG‐based microcrystals were also synthesized and used to fabricate PSCs (Figure S29). As summarized in Table S6, the PPG‐derived devices show the best performance, which may tentatively be attributed to the stronger interactions between PPG and α‐FAPbI_3_ compared to the other polymers. To examine whether a single‐step precursor route could achieve comparable performance, PSCs were also fabricated from perovskite films directly derived from FAI, PbI_2_, MACl, CsCl and PPG dissolved in DMF/DMSO. The resulting champion device has a substantially lower PCE of 23.46% (average 20.63%) compared with the microcrystal‐derived target devices (26.50%) (Figure S30 and Table S7).

The compatibility of PPG‐coated α‐FAPbI_3_ in the fabrication of p–i–n structured PSCs was also explored. The resulting p–i–n devices exhibit excellent photovoltaic performance, with a champion PCE of 26.33% (Figure S31 and Table S8). This high efficiency demonstrates that the approach is not limited to n–i–p configurations but is fully compatible with p–i–n device architectures.

#### 2.3.2 Defect Suppression Experiments

Space‐charge‐limited current (SCLC) measurements were conducted to assess the trap density in the perovskite films, using both electron‐only (FTO/SnO_2_/perovskite/PCBM/Au) and hole‐only (FTO/PEDOT: PSS/perovskite/spiro‐OMeTAD/Au) devices. The trap‐filled limit voltage (*V*_TFL_) of the electron‐only device decreases from 0.391 V in the control to 0.294 V in the target (Figure 3e). Similarly, the *V*_TFL_ for the hole‐only device also decreases from 0.084 V in the control to 0.056 V in the target (Figure S32). These results indicate that PPG effectively passivates charge defects in the target film [60, 61]. Transient photovoltage (TPV) measurements, conducted under open‐circuit conditions, show that the carrier recombination lifetime increases from 0.51 µs in the control to 0.68 µs in the target (Figure S33a), confirming reduced carrier recombination and trap density. Transient photocurrent (TPC) measurements, performed under short‐circuit conditions, yield carrier extraction times of 0.40 µs for the control device and 0.10 µs for the target device (Figure S33b), indicating enhanced carrier transfer efficiency in the target device. Furthermore, the target device exhibits a built‐in potential (*V*_bi_) value of 1.08 V (determined from the Mott–Schottky (M–S) plots), compared to 0.99 V observed for the control device (Figure S34). This increase in *V*_bi_ enhances the driving force for photo‐generated carrier separation, improving carrier transport. Defect passivation induced by PPG may be attributed to its structure with an abundance of ether oxygen atoms and terminal hydroxyl groups. Ether‐functionalized species interact strongly with undercoordinated Pb^2^^+^ ions on perovskite surfaces through the formation of Pb─O dative bonds, to effectively suppress trap states [62, 63, 64]. The enhanced *V*_OC_ of the target devices may be attributed to the remarkably low nonradiative recombination voltage loss [62, 65]. To further assess recombination in the PSCs, EQE of electroluminescence (EQE_EL_) measurements were performed. Notably, the target device has a higher EL intensity compared to the control device at the same bias voltage (Figure S35), achieving an EQE_EL_ of approximately 9.8% under an injection current of 26.03 mA/cm^2^, whereas the control device reaches only 1.2% EQE_EL_ under 25.45 mA/cm^2^ (Figure 3f). Nonradiative recombination voltage losses were calculated using Equation (1):

(1) $$V_{oc,loss}^{nonrad}=\frac{k_{B}T}{q}\;\ln\left(1/EQE_{EL}\right)$$

The target device has a lower nonradiative recombination voltage loss of 61 mV compared to the control device which has a value of 115 mV.

#### 2.3.3 Stability of the Films and PSCs

**FIGURE 4:**
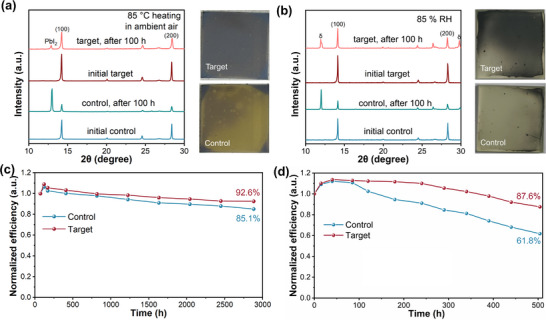
XRD patterns and photographs of the control and target perovskite films after accelerated aging under (a) thermal (85 °C in air) and (b) humidity (85% RH) stress conditions. (c) Stability of the unencapsulated control and target devices stored in the dark for 2880 hours. (d) Humidity stability test of the unencapsulated control and target devices conducted under conditions of approximately 65% RH and 25°C in air.

To evaluate the stability of the perovskite films, the films were exposed to accelerated aging under thermal (85 °C in air) and humidity (85% RH) stress conditions. After 100 hours at 85 °C, the control film degrades with extensive formation of PbI_2_ observed, denoted by the peak at 12.90° in the XRD pattern and the yellow color of the film (Figure 4a). In contrast, the target film largely retains its structure, although some PbI_2_ is observed. At high humidity, the control film also degrades after 100 hours to form predominantly δ‐FAPbI_3_, evidenced from the peak at 11.96° in the XRD pattern, whereas the amount of degradation in the target film is much reduced, although δ‐FAPbI_3_ is still observed (Figure 4b). Furthermore, after thermal aging at 160°C for 30 h, the I:Pb ratio of the target film remains at 2.91 (2.92 for the fresh film), which is higher than that of the control film (2.19 after aging and 2.67 for the fresh film), determined using XPS (Figure S36a,b), confirming improved iodine retention under elevated‐temperature conditions. The superior water stability of the target film is presumably due to its higher surface hydrophobicity, as confirmed by water contact angle measurements. The contact angle increases significantly from 53.1° in the control film (Figure S37a) to 69.8° in the target film (Figure S37b). The long‐term operational stability of both the control and target PSCs (unencapsulated) under low humidity conditions (RH = ∼1%) at 25°C was investigated. After 2880 hours, the control and target devices retain 85.1and 92.6% of their initial PCEs, respectively (Figure 4c). To assess thermal stability, PSCs were fabricated with the structure, FTO/SnO_2_/perovskite/PTAA/Au. After continuous heating at 85°C for 504 hours in a nitrogen glove box, the target device maintains approximately 77.1% of its initial PCE, whereas the control device retains only 55.8% (Figure S38). After storage for 504 hours at 65% RH, the control and target devices retain 61.8 and 87.6% of their initial PCEs, respectively (Figure 4d).

For the large‐scale production of PSCs, it would be advantageous if microcrystals used as starting materials exhibited long‐term stability during storage and transport. Based on this consideration, we investigated the influence of long‐term storage of microcrystals as raw materials on device performance. For this purpose, devices were fabricated in air using aged crystals and freshly prepared microcrystals. Without using PPG, a champion PSC device with a PCE of 24.44% was prepared from freshly prepared microcrystals, whereas that from aged microcrystals gave a champion PCE of only 21.54% (Figure S39). In contrast, when target microcrystals engineered with PPG were applied, the PCE of the fabricated devices showed only a minimal decrease from 26.50% (using freshly target microcrystals) to 26.04% (using aged target microcrystals) (Figure S39). For statistical analysis, 30 devices were fabricated using 6‐month‐aged control microcrystals, and another 30 from those using 6‐month‐aged target microcrystals. The average PCE of devices derived from aged control microcrystals is 20.5% with a large distribution range, whereas an average PCE of 25.64% with a much narrow distribution range was achieved when aged target microcrystals were employed in the fabrication process (Figure S40).

## 3 Conclusions

We developed a scalable approach that uses PPG in the ITC process from low‐cost PbI_2_ to form highly stable, phase‐pure target microcrystals that maintain their integrity for over six months in ambient air. Almost 50 g of the target microcrystals were prepared in a single batch, which is sufficient to fabricate modules with an area of ca. 23 m^2^. Redissolving these microcrystals and using the solution to generate perovskite films in air yields highly uniform films with reduced defect densities. The perovskite films were evaluated in PSCs with the champion target device achieving a PCE of 26.50% (certified 26.22%) with an enhanced *V*_OC_ of 1.214 V. Furthermore, leveraging the uniformity and high crystal quality of large‐area films, we prepared a perovskite solar module with an aperture area of 28.99 cm^2^ that achieved a PCE of 22.66%. Notably, unencapsulated target devices retain over 92.6% of their initial PCE after 2880 hours without encapsulation under 1% relative humidity at 25°C, demonstrating their excellent long‐term stability. This work provides a simple and efficient route toward efficient, stable, and scalable perovskite photovoltaics.

## Conflicts of Interest

The authors declare no conflicts of interest.

## Supporting information




**Supporting File 1**: adma72425‐sup‐0001‐SuppMat.docx.

**Supporting File 2**: adma72425‐sup‐0002‐Data.zip.

## Data Availability

The data that support the findings of this study are available from the corresponding author upon reasonable request.
