# Supplementary material for: Scalable Fabrication of High‐Efficiency Thin‐Film Perovskite Solar Cells in Air via Polymer‐Mediated Synthesis of α‐FAPbI3 Microcrystals
Source: Adv Mater. 2026 Feb 5;38(14):e22508. doi: 10.1002/adma.202522508 (PMC12966973; doi:10.1002/adma.202522508)
Supplement: Supplementary file 1 — Supporting File 1: adma72425‐sup‐0001‐SuppMat.docx. [file ADMA-38-e22508-s002.docx]

Supplementary Information for

**Scalable Fabrication of High-Efficiency Thin-Film Perovskite Solar Cells in Air via Polymer-Mediated Synthesis of α-FAPbI_3_ Microcrystals**

Fan Shen^1,2,3#^, Chenxu Zhao^3#^, Jia Xu^1,2#^, Xunhui Wang^3#^, Jiale Chen^1,2^, Huijing Liu^1,2^, Pengchen Zou^1,2,3^, Xuewei Liu^1,2^, Yao Fu^1,2^, Huifang Han^1,2^, Kun Lang^1,2^, Yijun Wang^1,2^, Xingyu Gao^4^, Zhaofu Fei^3*^, Hong Zhang^5*^, Paul J. Dyson^3*^, Jianxi Yao^1,2*^

^1^ New Energy Generation National Engineering Research Center, North China Electric Power University, Beijing 102206, China.

^2^ Beijing Key Laboratory of Energy Safety and Clean Utilization, North China Electric Power University, Beijing 102206, P. R. China.

^3^ Institute of Chemical Sciences and Engineering, École Polytechnique Fédérale de Lausanne (EPFL), Lausanne 1015, Switzerland.

^4^ Shanghai Synchrotron Radiation Facility (SSRF), Shanghai Advanced Research Institute, Chinese Academy of Sciences, No. 239 Zhangheng Road, Shanghai 201204, China.

^5^ State Key Laboratory of Photovoltaic Science and Technology, Shanghai Frontiers Science Research Base of Intelligent Optoelectronics and Perception, Institute of Optoelectronics, College of Future Information Technology, Fudan University, Shanghai 200433, China.

^#^These authors contributed equally to this work.

*Corresponding authors: [hzhangioe@fudan.edu.cn](mailto:hzhangioe@fudan.edu.cn); [zhaofu.fei@epfl.ch](mailto:zhaofu.fei@epfl.ch); [paul.dyson@epfl.ch](mailto:paul.dyson@epfl.ch); [jianxiyao@ncepu.edu.cn](mailto:jianxiyao@ncepu.edu.cn).

**Materials and Methods**

**Materials:**

SnO_2_ colloid precursor (tin(IV) oxide, 15% in H_2_O colloidal dispersion), 2-methoxyethanol (2-ME), N,N-dimethylformamide (DMF), dimethyl sulfoxide (DMSO), chlorobenzene (CB), isopropanol (IPA), and cesium chloride (CsCl) were purchased from Alfa Aesar. Cyclohexylmethylammonium iodide (CHMAI) was purchased from TCI. Lead (II) iodide (PbI_2_) (99%), formamidinium iodide (FAI), methylamine chloride (MACl), 2,2′,7,7′-tetrakis[N,N-di(4-methoxyphenyl)amino]1−9,9′-spirobifluorene (Spiro-OMeTAD), and poly[bis(4-phenyl) (2,4,6-trimethylphenyl) amine] (PTAA) were purchased from Xi’an Polymer Light Technology (China). 4-Tert-butyl pyridine (TBP) and lithium-bis(trifluoromethanesulfonyl)imide (Li-TFSI) were acquired from Sigma-Aldrich. Polypropylene glycol (PPG), polyethylene glycol (PEG), and polyvinyl alcohol (PVA) were purchased from Innochem.

**Synthesis of α-FAPbI_3_ microcrystals:**

PbI_2_ (3.6880g, 8.0 mmol), FAI (1.3757g, 8.0 mmol), and PPG (0.46 mg, 0.008 mol) were mixed in 2-ME (10 mL). The yellow pre-precursor solution was heated at 120 °C under stirring for 1 h, during which time black α-FAPbI_3_ microcrystals formed.^1^ The resulting α-FAPbI_3_ microcrystals were collected by filtration and heated at 150 °C for 30 min in a vacuum oven. Yield: 4.0603g (80.2%). α-FAPbI_3_ microcrystals using 0%, 0.5, and 1.5% molar ratios of PPG relative to PbI_2_ were prepared from the same amount of PbI_2_ (3.6880g, 8.0 mmol), FAI (1.3757g, 8.0 mmol), and the corresponding PPG (0 mg, 0.23mg, 0.70 mg). The yields were 4.25 g (80.1%), 4.290 g (80.8%), 4.380g (81.1%), respectively.

**Synthesis of α-FAPbI_3_ microcrystals on a 48.50 g scale:**

PbI_2_ (36.90 g, 80 mmol), FAI (13.77 g, 80 mmol), and PPG (1.0% molar ratio to PbI_2_) were dispersed in 100 mL of 2-ME. The yellow pre-precursor solution was heated and stirred at 120 °C for 1 h, during which time α-FAPbI_3_ microcrystals formed. After filtration, 41.0 g of α-FAPbI_3_ microcrystals were obtained. From the filtrate, another portion of 7.50 g was obtained by concentration of the solvent (from 100 mL to 20 mL) followed by subsequent filtration—total yield: 48.50 g (95.8%).

**Note S1:**

**Estimation of the area of perovskite solar modules available from 48.5 g α-FAPbI_3_ microcrystals:**

Assuming the thickness of the perovskite layer is 500 nm, which is an average of the modules,^2-4^ the total volume of 1 cm^-2^ PSCs will be 1.0 x 1.0 x 0.00005 = 5 x 10^-5^ cm^3^. The density of the α-FAPbI_3_ microcrystals is 4.10 g/cm^3^. The weight of α-FAPbI_3_ per cm^-3^ is: 5x10^-5^ x 4.1 = 2.05 x 10^-4^ g. The total area of modules from 48.50 g of α-FAPbI_3_ microcrystals will be 48.50 g/2.05x10^-4^ = 236600 cm^-2^ (23.66 m^2^). Considering the standard size of silicon modules is 21 x 21 cm^2^, the total number of modules of 441 cm^2^ will be 236600/441 = 536.

**Fabrication of perovskite films and devices:**

FTO glass substrates were cleaned by sequentially washing with detergent, deionized water, and ethanol, and further cleaned with UV ozone for 30 min directly before use. The substrates were spin-coated with a SnO_2_ nanoparticle film (diluted with deionized water to 4 wt %) at 4000 rpm for 20 s, then annealed at 185 °C on a hot plate for 30 min in ambient air. The FTO/SnO_2_ substrates were cleaned with UV ozone for 30 min. 1.66 M stock precursor solutions containing α-FAPbI_3_ microcrystals (44.65 g, 0.70 mmol), MACl (0.95 g, 0.14 mmol) (20% mol), and CsCl (0.59 g, 0.035 mmol) (5 mol%) in DMF/DMSO (4.25 mL, 7.5:1, v/v) were prepared and used for the subsequent experiments. The precursor solution was deposited on the SnO_2_-coated FTO substrates by spin coating at 1000 and 5000 rpm for 5 and 20 s, respectively, and 800 µL of diethyl ether was added. The films were annealed at 120 °C for 1 h, and then CHMAI (6 mg mL^-1^ in IPA) was spin-coated onto the films at 6000 rpm without any further processing. Next, 50 µL spiro-OMeTAD solution, which consisted of 60 mg spiro-OMeTAD, 25.5 µL TBP, 15.5 µL Li-TFSI solution (520 mg mL^-1^ in acetonitrile) in 0.7 mL chlorobenzene, was spin-coated at 4000 rpm for 30 s. For the thermal stability tests, PTAA was used instead of spiro-OMeTAD as the hole transport layer. A 30 mg mL⁻¹ PTAA solution in toluene was spin-coated at 2000 rpm for 30 s.^5-7^. Finally, an 80 nm Au layer was thermally evaporated onto the films. The entire fabrication process was conducted in air with the temperature ranging from 20-30 °C and a humidity of ~30%.

**Fabrication of the perovskite solar module:**

A PSM with 8 sub-cells connected in series was fabricated on FTO glass substrates measuring 65×70 mm^2^. The series interconnection was achieved using P1, P2, and P3 lines patterned with a laser scribing technique. The procedure for preparing PSMs using the spin-coating process was the same as that described for PSCs, with the exception that, after spin coating, the perovskite film was subjected to vacuum flashing.

The FTO substrate was pre-patterned for P1 (width 40 μm) at a power of 1.8 W with a speed of 300 mm/s and a frequency of 500 kHz. The P2 lines (width 150 μm) were patterned with an average laser power of 0.33 W at a speed of 500 mm/s and a frequency of 500 kHz. The P3 line (width 40 μm) was made using the same scribing conditions as the P2 line. The distance between P1 and P3 is around 210 μm, and the geometric fill factor (GFF) is around 96.7%.

**Note S2:**

**First principles calculations:**

All calculations were performed with the Vienna ab initio Simulation Package (VASP)^8^ within the frame of density functional theory (DFT). The exchange-correlation interactions of electrons were described via the generalized gradient approximation (GGA) with the PBE functional^9^, and the projector augmented wave (PAW) method^10^ was used to describe the interactions of electrons and ions. Additionally, the DFT-D3 method^11, 12^ was used to account for the long-range van der Waals forces present within the system. The Monkhorst-Pack scheme^13^ was used for the integration in the irreducible Brillouin zone. A kinetic energy cutoff of 450 eV was chosen for the plane-wave expansion. The lattice parameters and ionic position were fully relaxed, and the total energy was converged within 10^-5^ eV per formula unit. The final forces on all ions are less than 0.02/Å.

**Characterization:**

X-ray photoelectron spectroscopy (XPS) and ultraviolet photoelectron spectroscopy (UPS) were performed using an ESCALAB 250Xi instrument (Thermo Fisher Scientific). Scanning electron microscopy (SEM) images were acquired using a SU8010 SEM (Hitachi, Chiyoda City, Japan, 3.0 kV, 10100 nA). Atomic force microscopy (AFM) was conducted on a Bruker multimode atomic force microscope. X-ray diffraction (XRD) patterns were obtained on a SmartLab X-ray diffractometer (Rigaku) using Cu Kα radiation (λ=1.5418Å). Grazing incident X-ray diffraction (GIXRD) patterns were acquired using a Smartlab XRD employing the 2θ-Sin^2^ (*ψ*) method at an incident angle of 0.2° with different tilt angles ψ. In situ grazing incidence wide-angle X-ray scattering (GIWAXS) was conducted at the BL14B1 beamline at the Shanghai Synchrotron Radiation Facility (SSRF). The X-ray beam wavelength was 1.24 Å with an energy of 10 KeV, and the incident angle was 0.3°. In situ photoluminescence (PL) spectra were recorded using a QE-Pro spectrometer (Ocean Optics), operating in kinetic mode with an integration time of 1 s for each spectrum. Dynamic light scattering (DLS) was acquired using a Zetasizer Nano ZS90 instrument. UV-vis spectra were recorded on a UV-2450 spectrophotometer (Shimadzu). Time-of-flight secondary ion mass spectrometry (TOF-SIMS) was performed using a TOF-SIMS 5-100 (ION-TOF GmbH, Germany). Steady-state PL spectra and time-resolved TRPL spectra were acquired using a FLS1000 spectrometer (Edinburgh Instruments, Livingston, UK). Transient photocurrent (TPC) and transient photovoltage (TPV) measurements were performed on a transient photocurrent and photovoltage measurement system (TranPVC-M, Oriental Spectra Technology (Guangzhou) Co., Ltd.). Electrochemical impedance spectroscopy (EIS) measurements and Mott-Schottky analysis were obtained using a potentiometer (CHI604E, CH instruments) under dark conditions. Current density-voltage (*J*–*V*) curves were measured using a Keithley 2400 Source Meter under simulated one-sun AM 1.5G illumination (100 mW cm^-2^) with a sunlight simulator (XES-300T1, SAN-EI Electric, AM 1.5G 100 mW cm^-2^), and the light intensity was calibrated with a standard silicon reference cell. The devices were masked with an area of 0.06 cm^2^ during testing. The sweeping parameters for reverse scanning were 1.3 V→ –0.2 V, step 0.02 V and for forward scanning were –0.2 V→1.3 V, step 0.02 V.

**Statistical Analysis:**

All statistics analyses were performed with OriginPro software. All the data keep at least two significant digits after the decimal points by the rounding-off method. The data obtained from XPS, UPS, XRD, DLS, in-situ GIWAXS, in-situ PL, SEM, AFM, GIXRD, PL, confocal PL mapping, TOF-SIMS, *J*−*V*, MPPT, EQE, EQE_EL_ were the original data without normalization. The other data were obtained by transferring the corresponding original data according to the calculation formula. The grain statistics data of SEM were calculated using the Nano Measurer software. Biexponential decay function was applied to TRPL decays to infer the carrier extraction/recombination dynamics. The statistical distribution data of PCE was got from 30 independent devices.


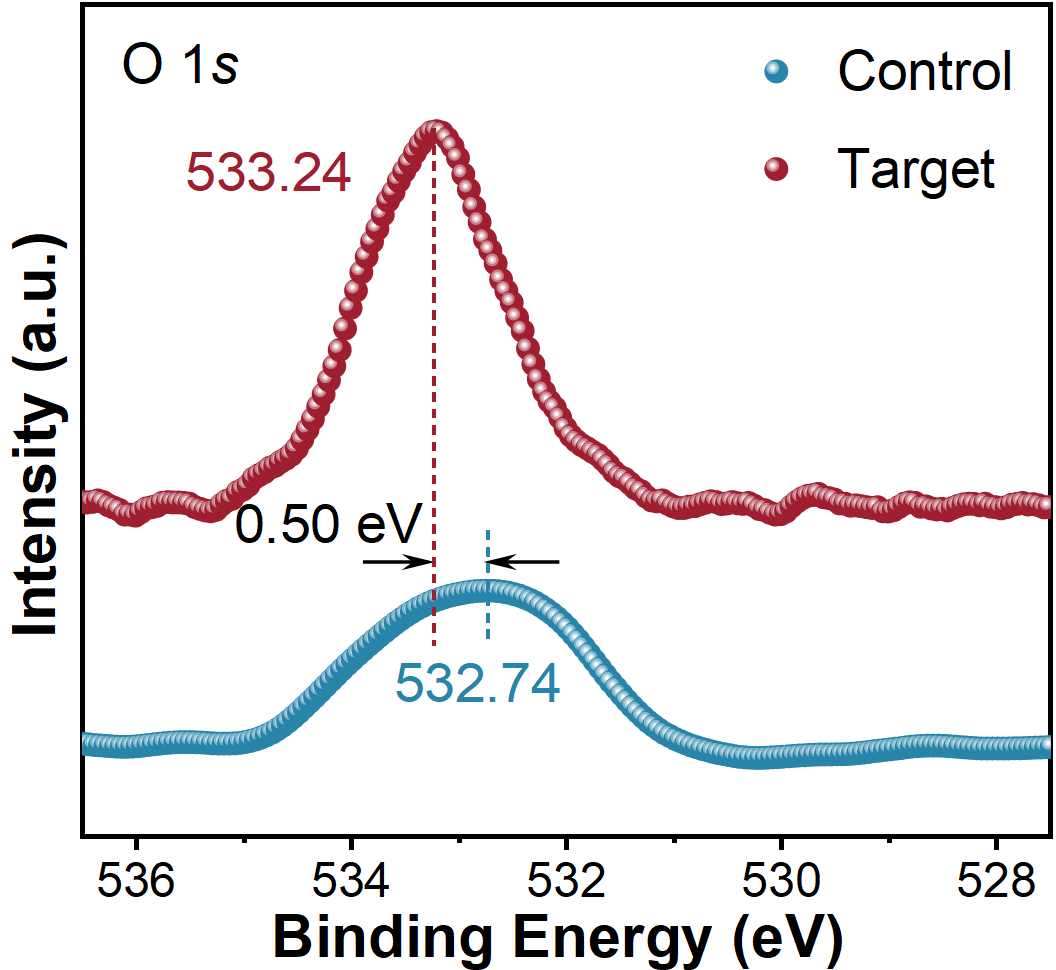


**Figure S1.** X-ray photoelectron spectroscopy (XPS) analysis of O 1*s* for the perovskite microcrystals.


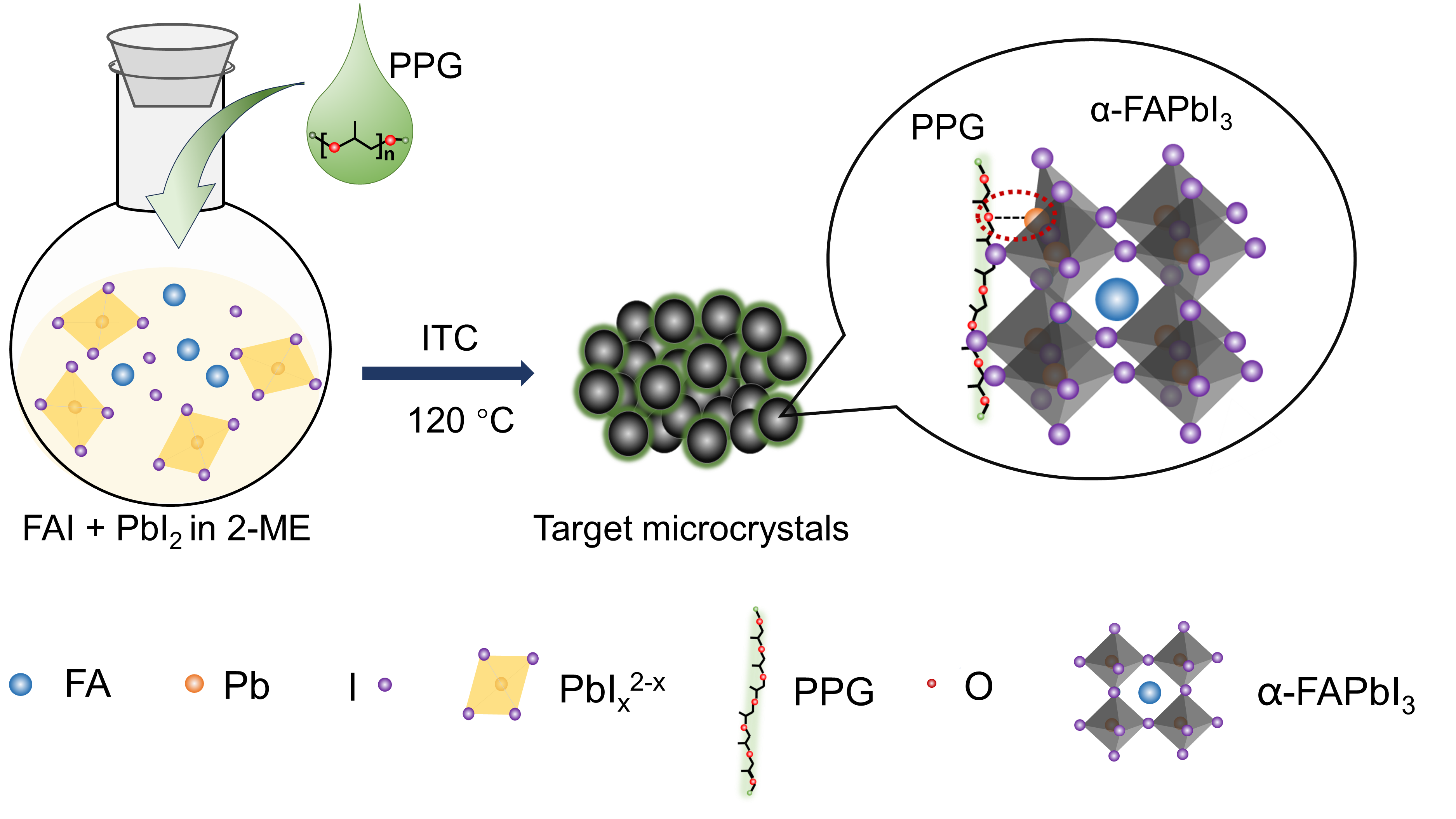


**Figure S2.** Schematic highlighting the interaction between PPG and the α-FAPbI_3_ microcrystals.


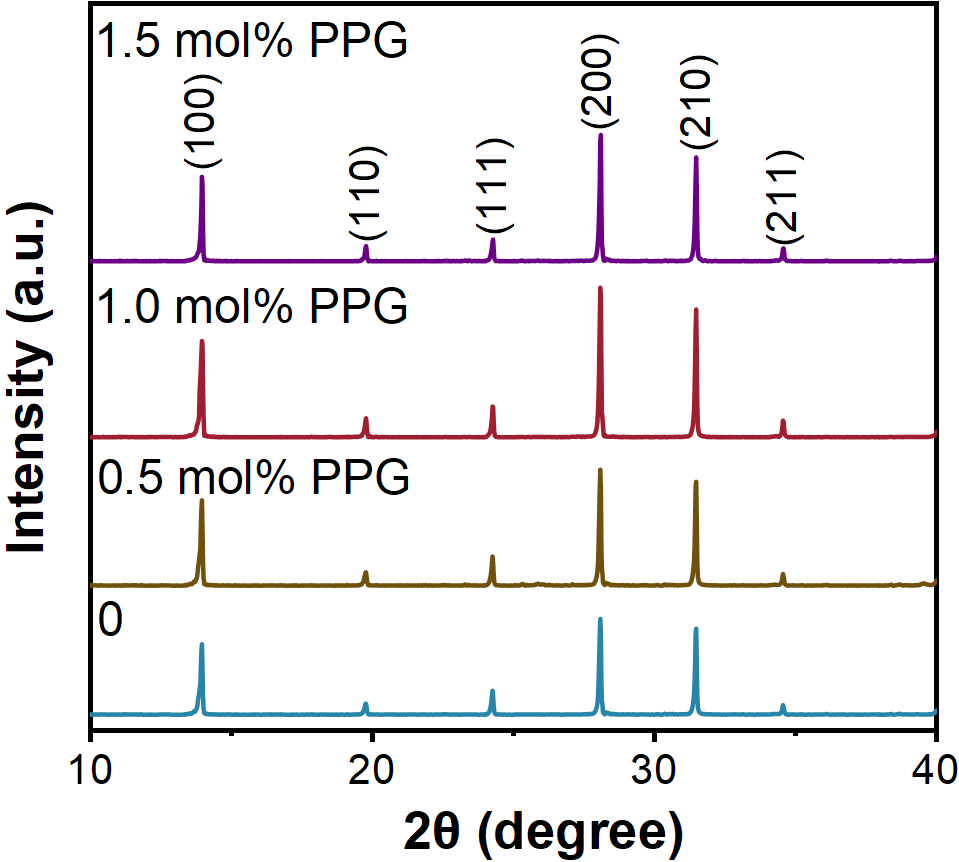


**Figure S3.** X-ray Diffraction (XRD) patterns of the perovskite microcrystals with different concentrations of PPG.


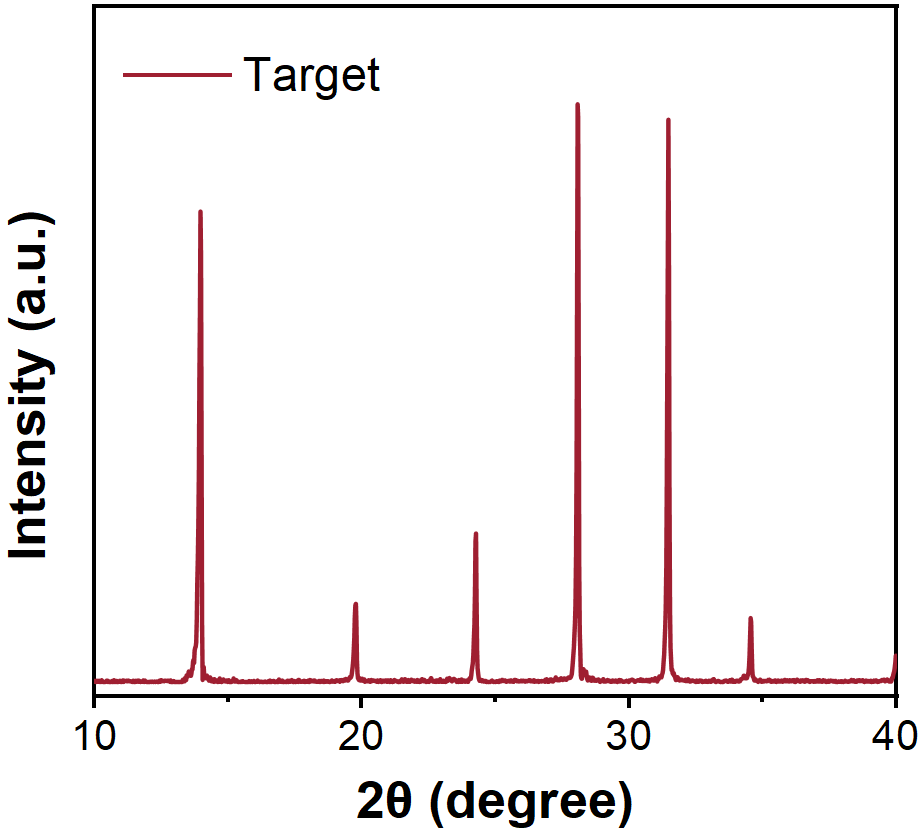


**Figure S4.** XRD pattern of the target microcrystals prepared at 25 °C under high-humidity conditions (~60% RH).


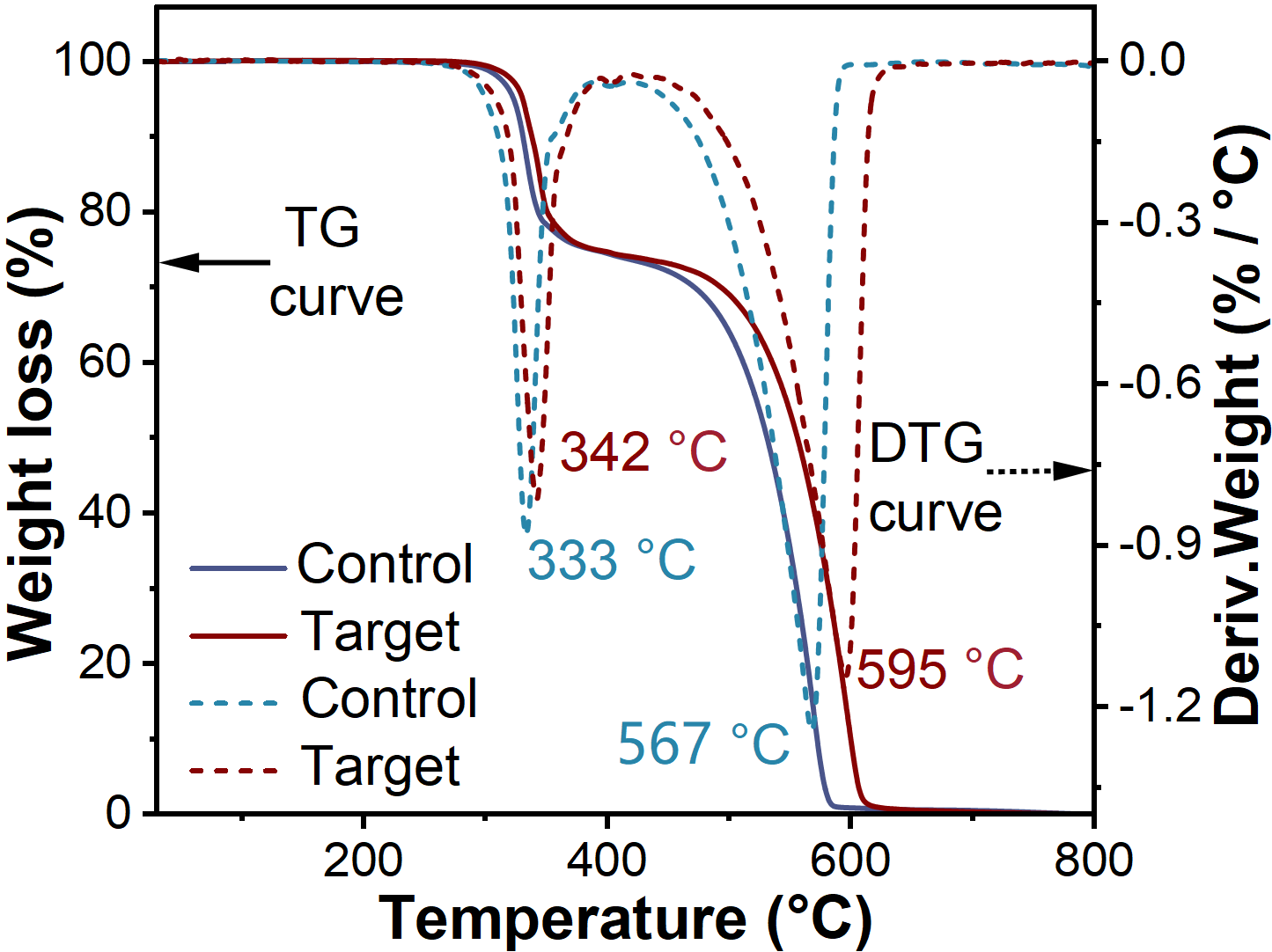


**Figure S5.** Thermogravimetric (TG) and derivative thermogravimetric (DTG) curves of the control and target microcrystals.


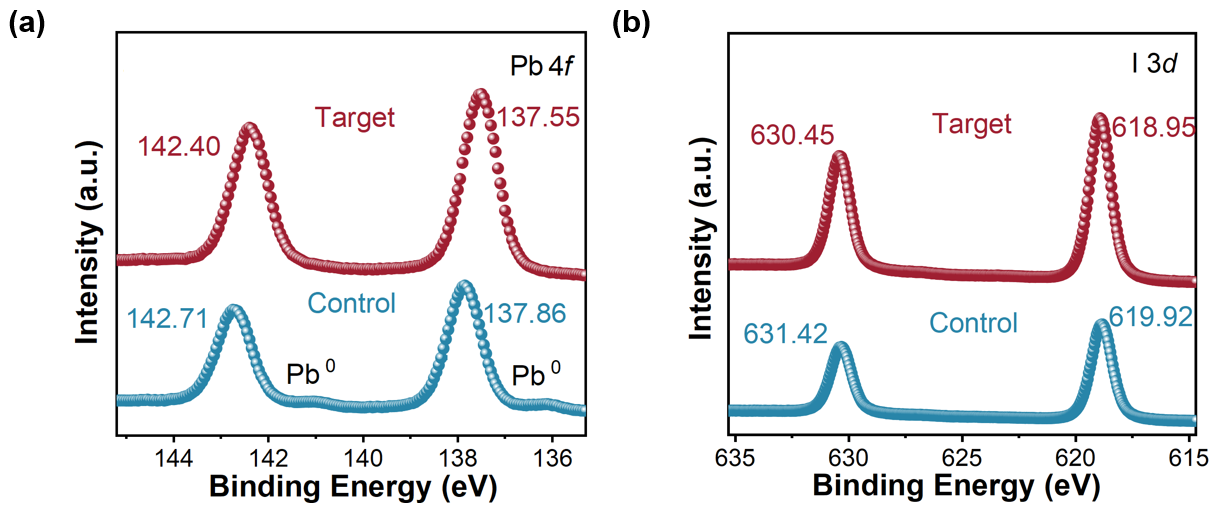


**Figure S6**. XPS spectra of (a) Pb 4*f* and (b) I 3*d* for the control and target microcrystals after 6 months of storage in air (25 °C, 40–50% relative humidity).


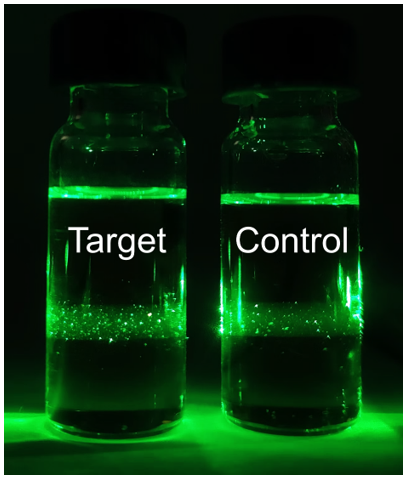


**Figure S7.** Photograph showing the Tyndall effect of the precursor solutions (illuminated with 532 nm laser).


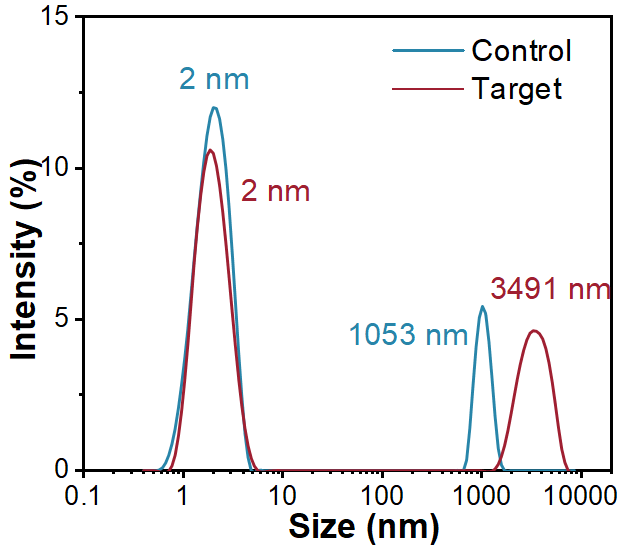


**Figure S8.** Dynamic light scattering (DLS) spectra of the perovskite precursor solutions.


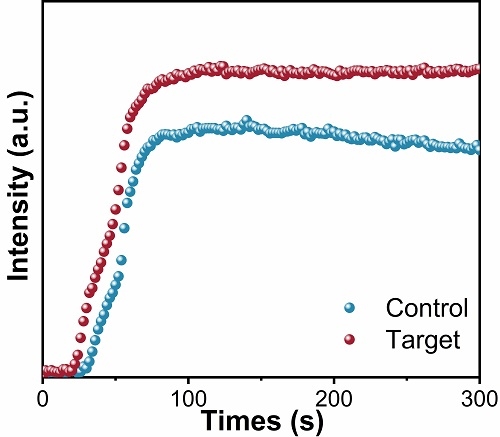


**Figure S9.** Time-resolved integrated peak area intensity for α-FAPbI_3_ from **Figure 2** (a) and (b).


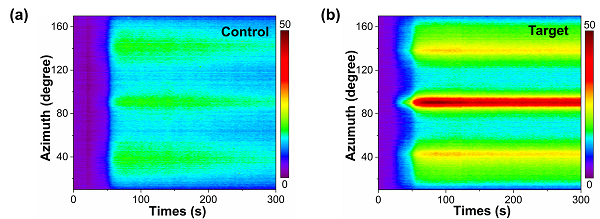


**Figure S10.** Evolution of the azimuth angle along the (100) planes of α-FAPbI_3_ for the (a) control and (b) target films.


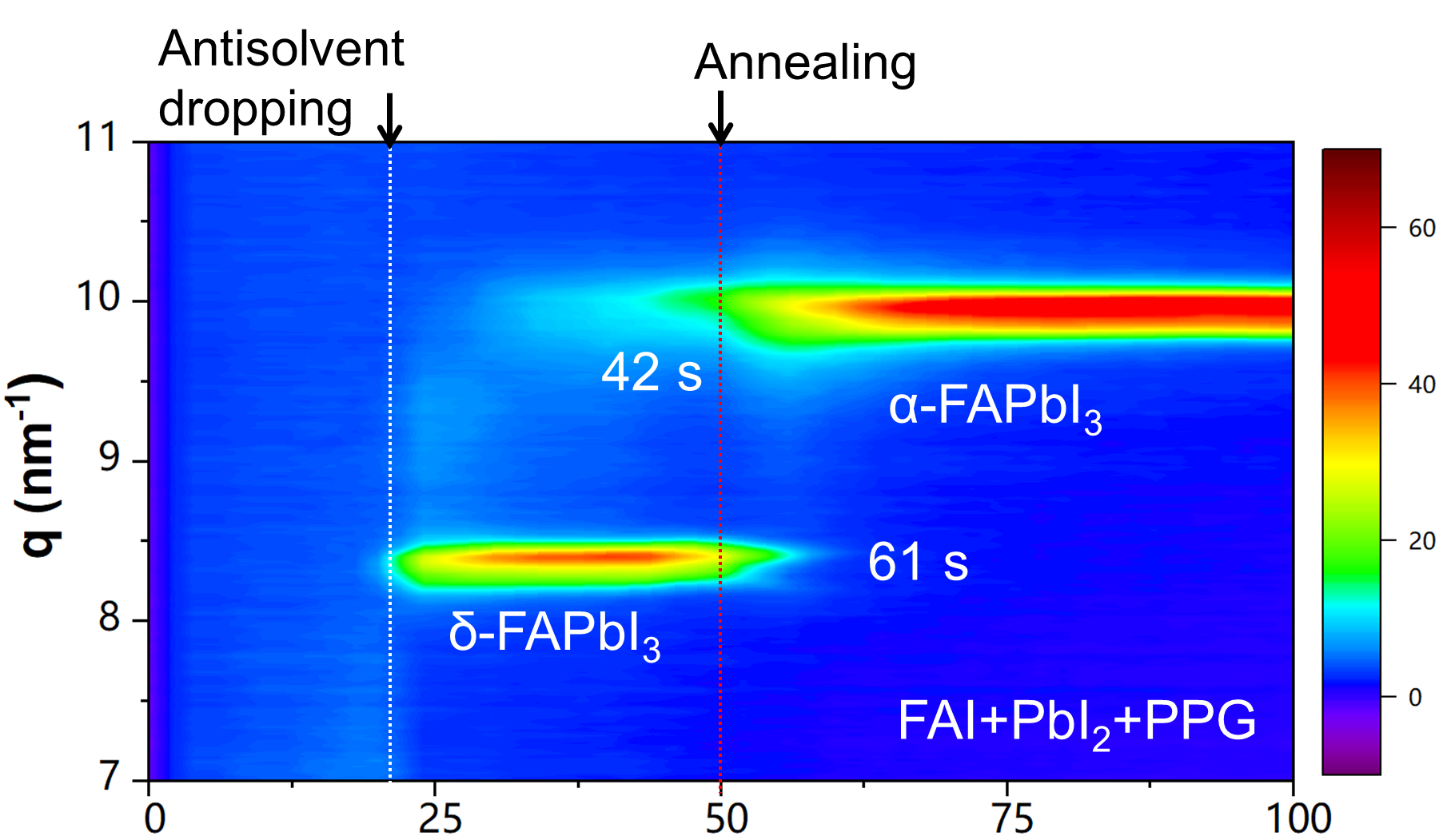


**Figure S11.** In situ GIWAXS spectra obtained during the crystallization of a perovskite film prepared by directly mixing FAI, PbI_2_ and PPG in DMF/DMSO.


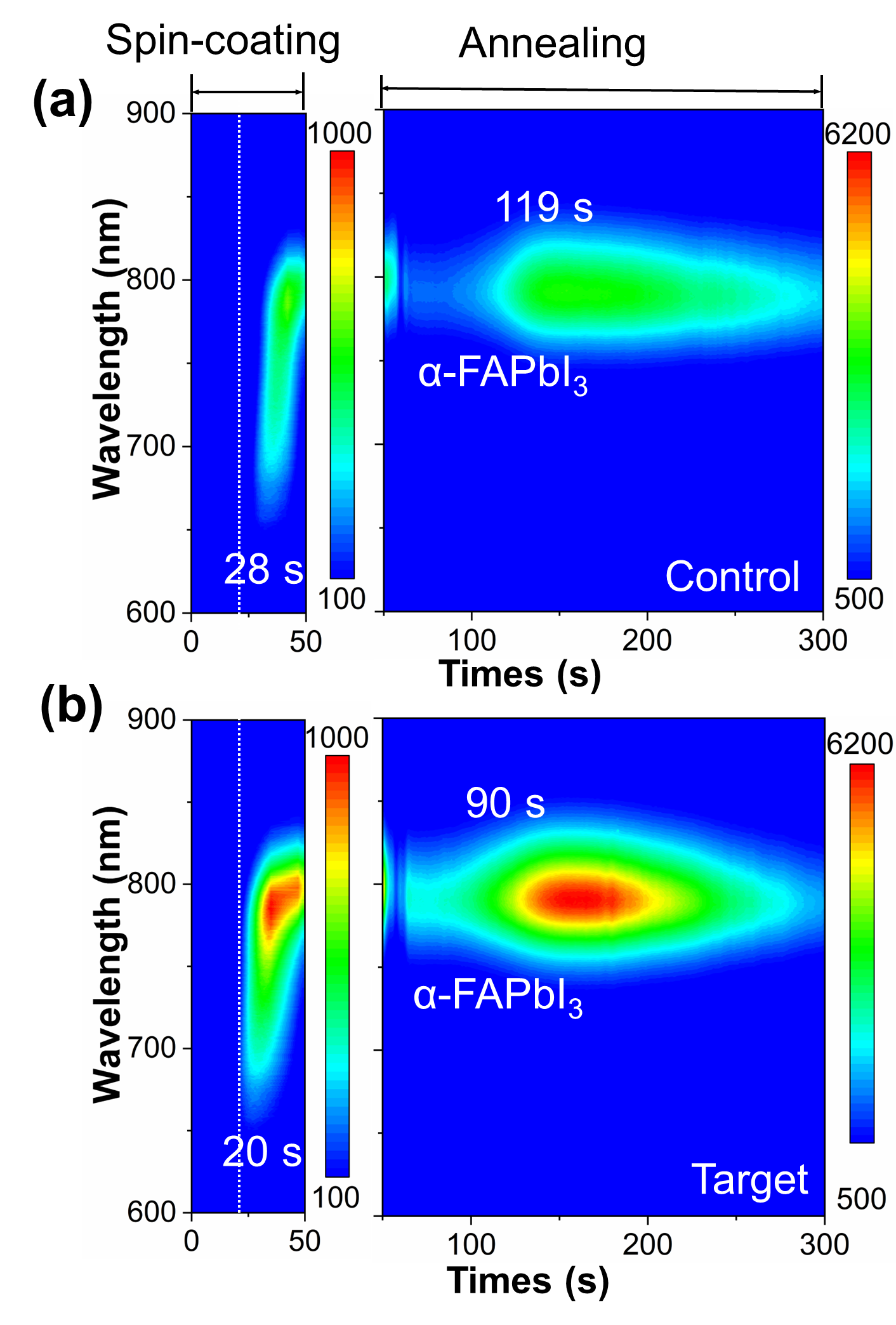


**Figure** **S12.** In situ photoluminescence (PL) spectra during spin-coating and annealing of the (a) control and (b) target films.


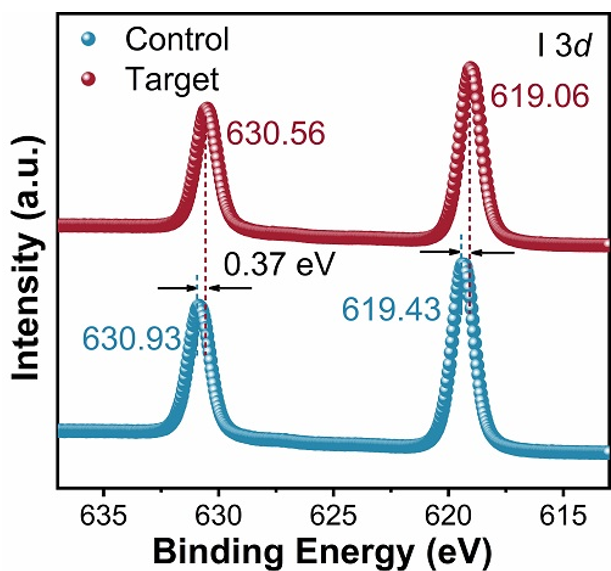


**Figure S13.** XPS I 3*d* spectra of the control and target films.


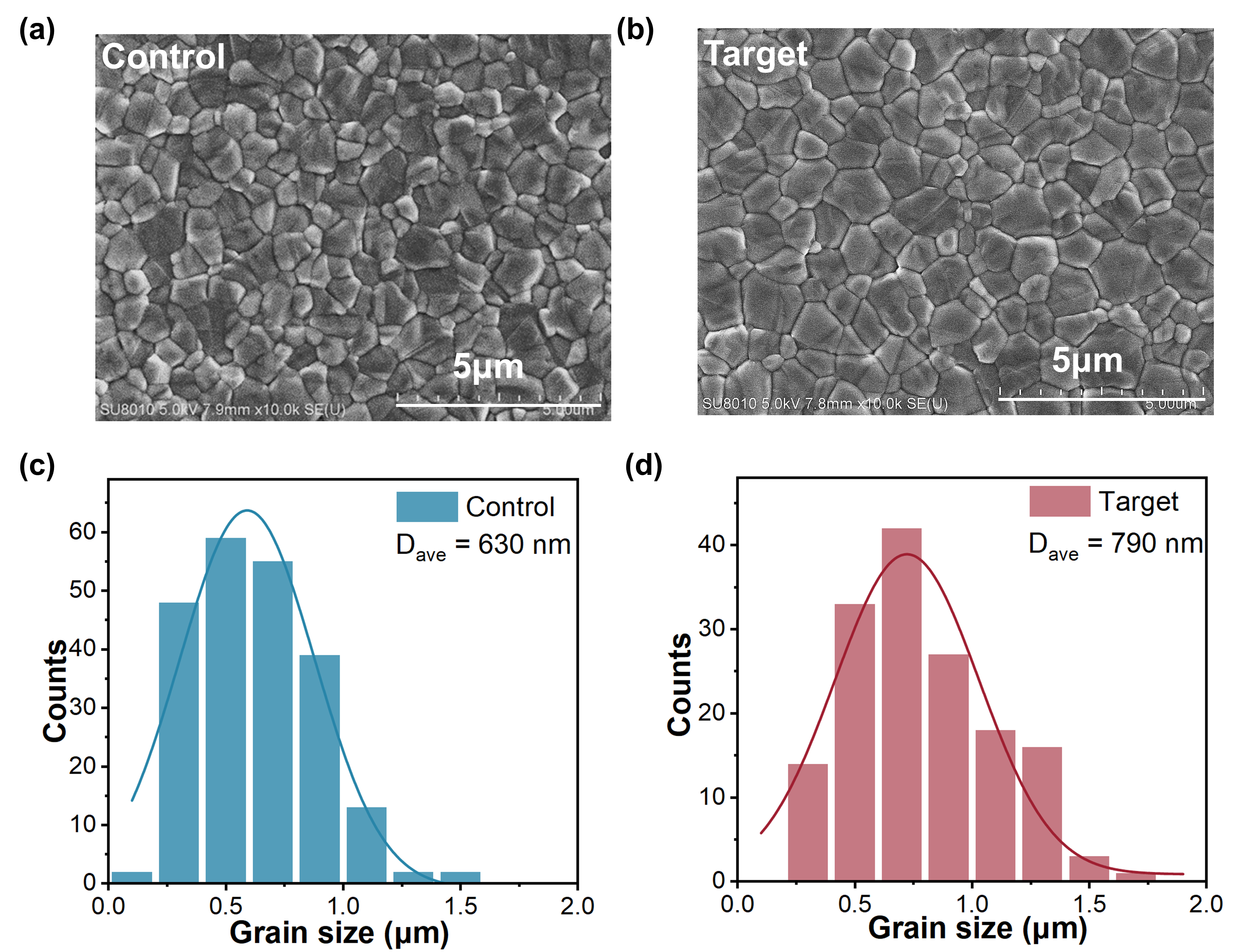


**Figure S14.** Top-view scanning electron microscopy (SEM) images of the (a) control and (b) target films. Grain size distributions of the (c) control and (d) target films.


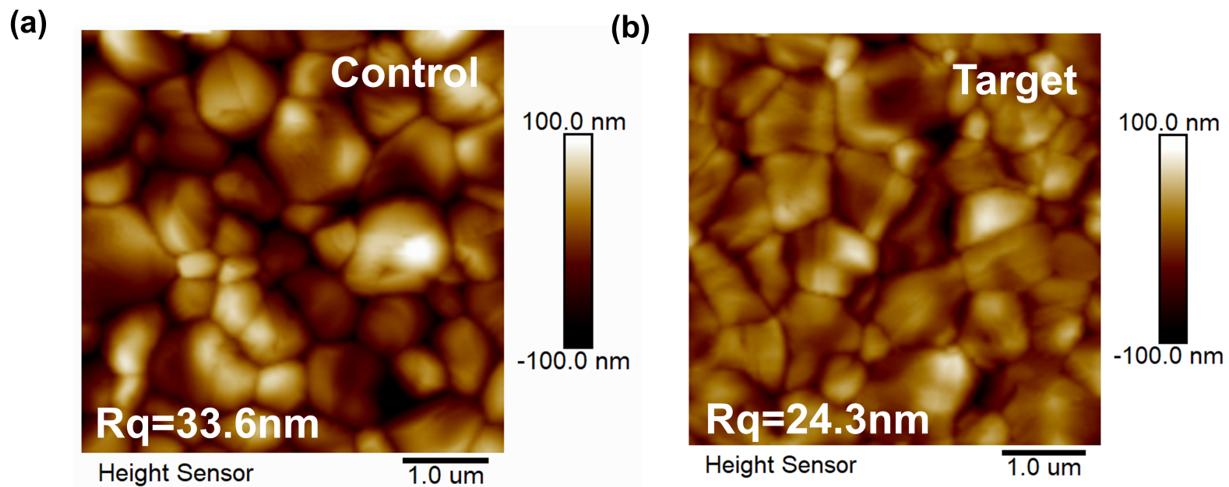


**Figure S15.** Atomic force microscopy (AFM) images of the (a) control and (b) target films.

**
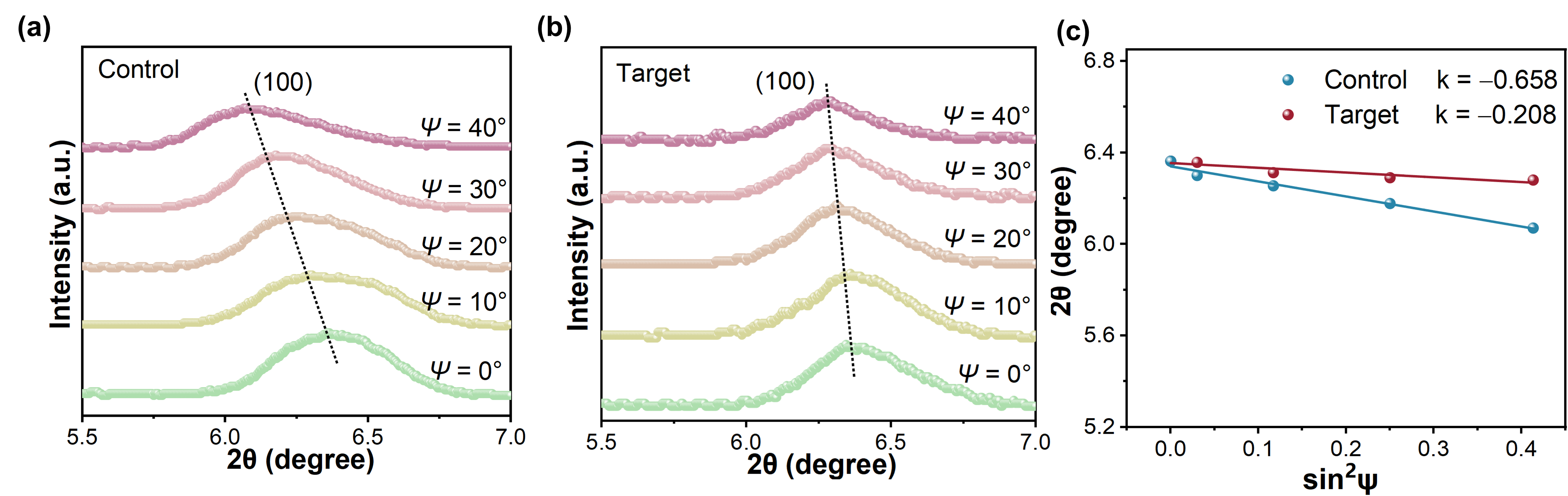
**

**Figure S16.** Grazing incidence X-ray diffraction (GIXRD) patterns at varying tilt angles (0 to 40°) for the (a) control and (b) target films. (c) Linear fit relationship of 2θ-sin^2^ (ψ) of the control and target films.


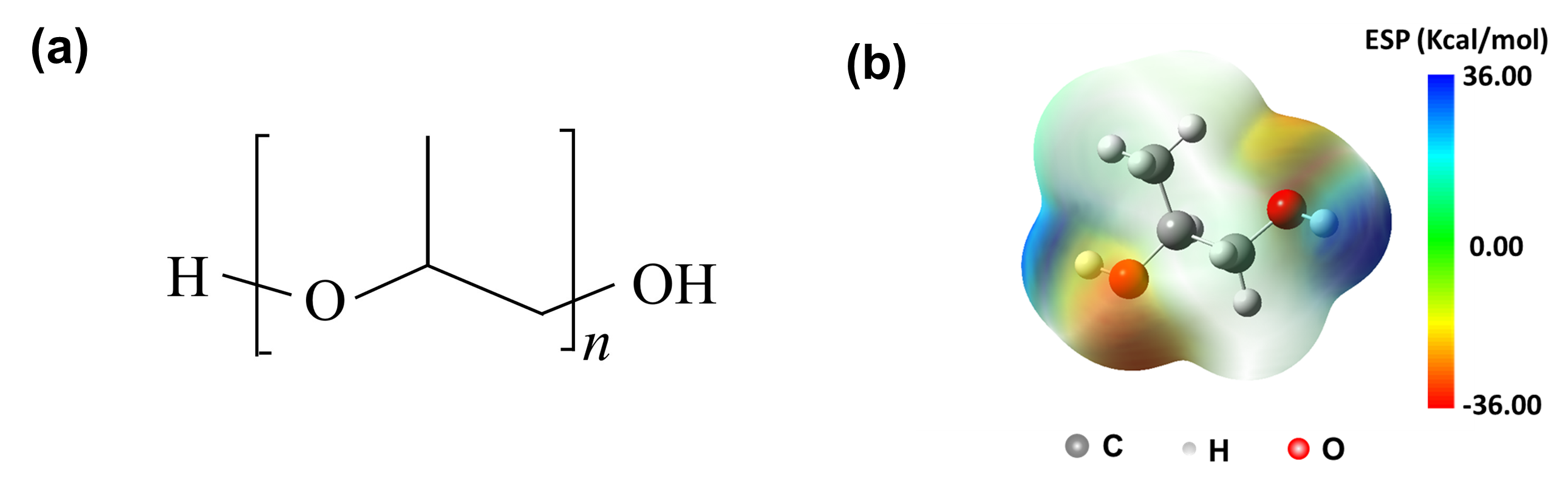


**Figure S17.** (a) Molecular structure of PPG. (b) Electrostatic surface potential distribution of the PPG monomer.


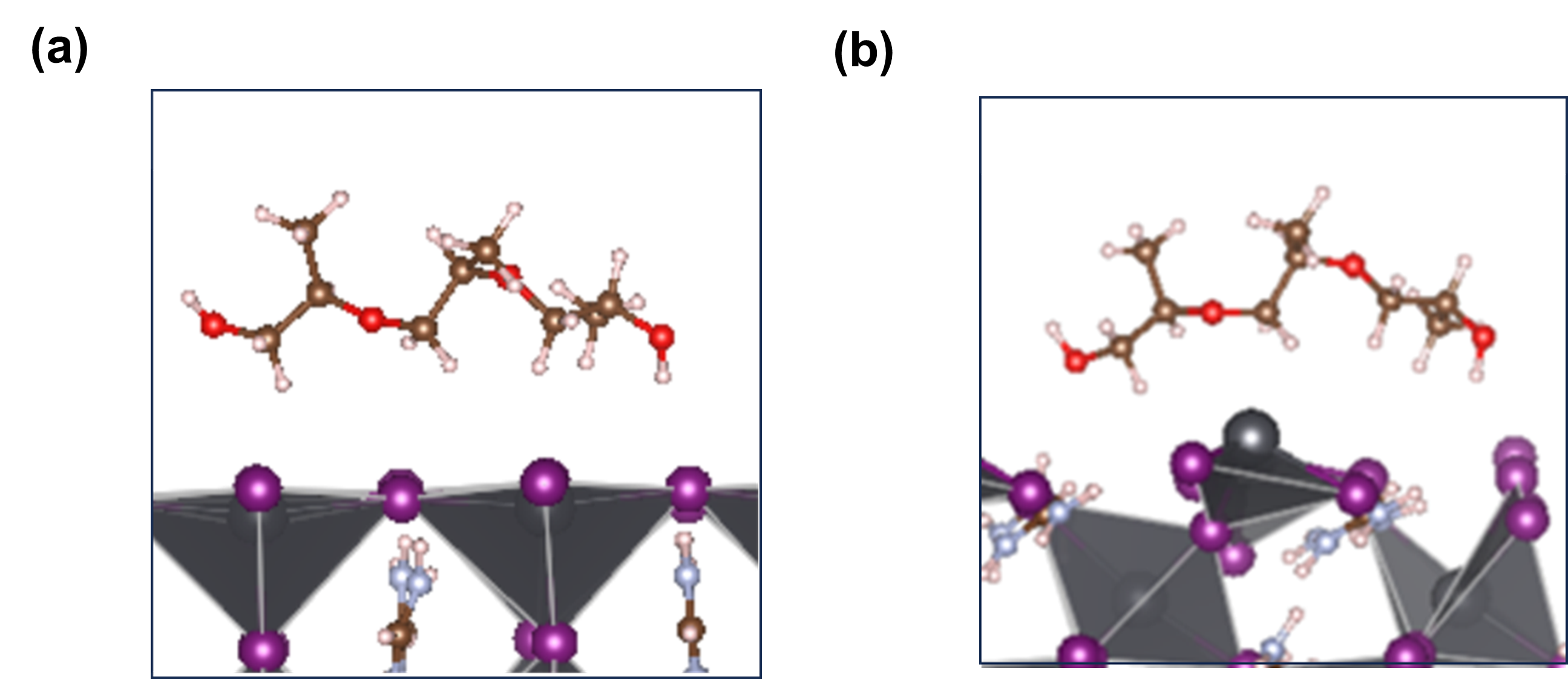


**Figure S18.** Effect of PPG on the surface energy of α-FAPbI_3_ for the (a) (100) plane and (b) (111) plane.


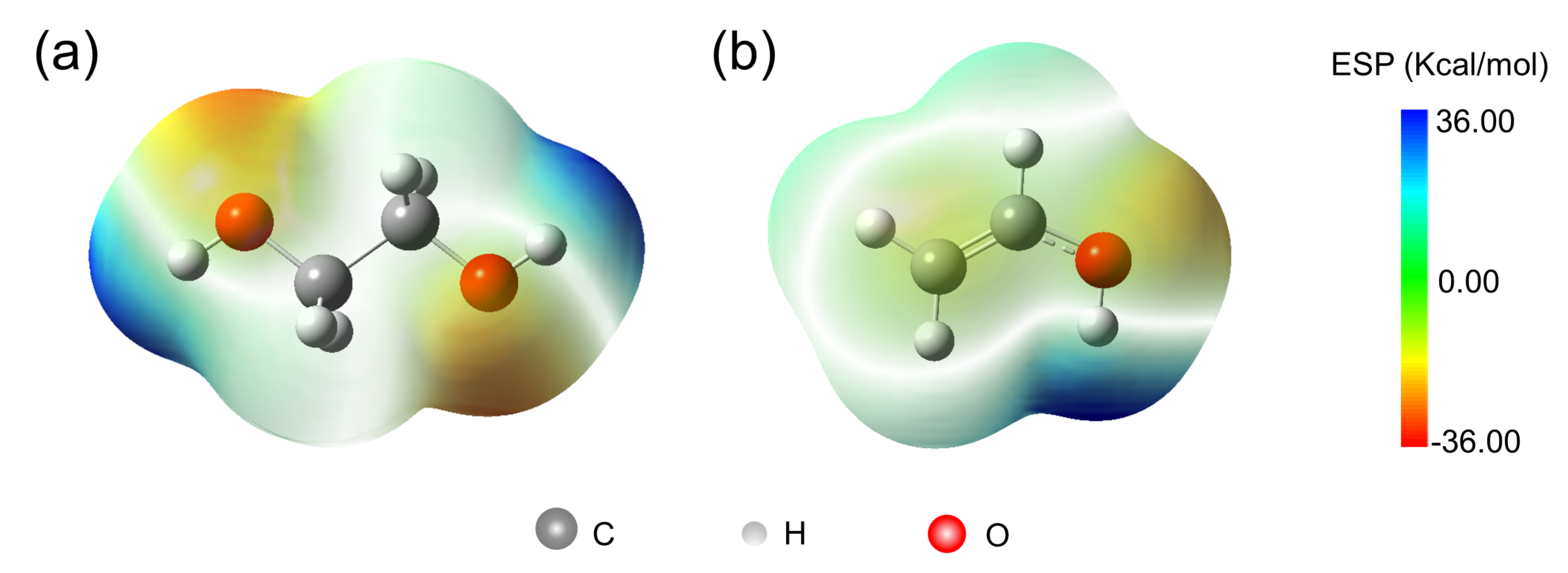


**Figure S19**. Electrostatic surface potential distribution of the (a) PEG monomer and (b) PVA monomer.


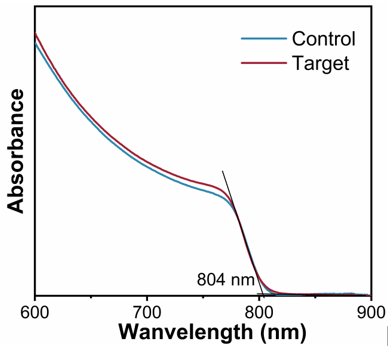


**Figure S20.** Ultraviolet-visible (UV-Vis) absorption spectra of the control and target films.


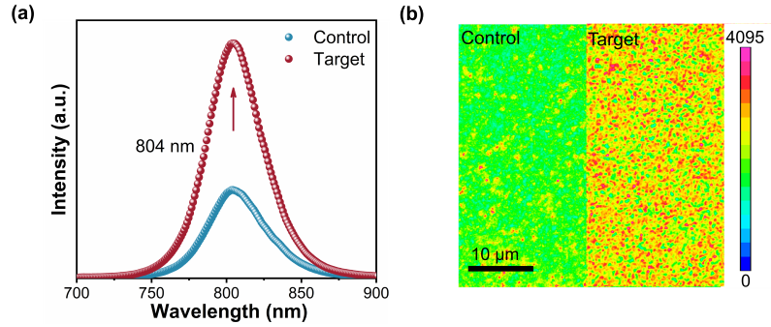


**Figure S21.** (a) Steady-state PL spectra and (b) confocal PL mapping of the perovskite films deposited on glass substrates.


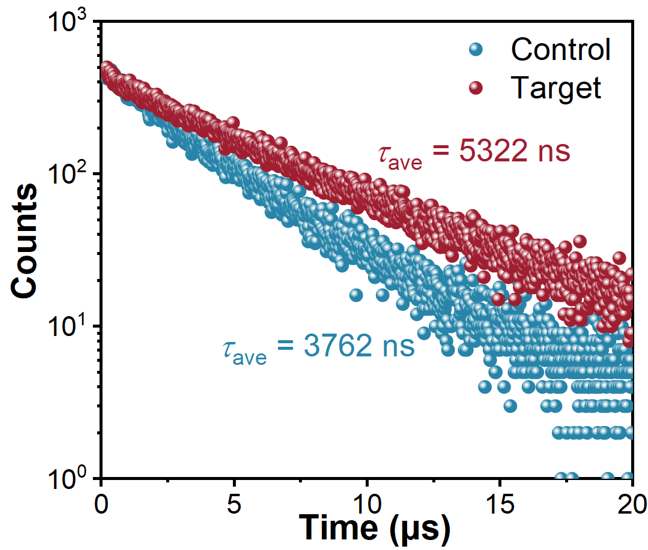


**Figure S22.** Time-resolved photoluminescence (TRPL) spectra of the perovskite films deposited on glass substrates.


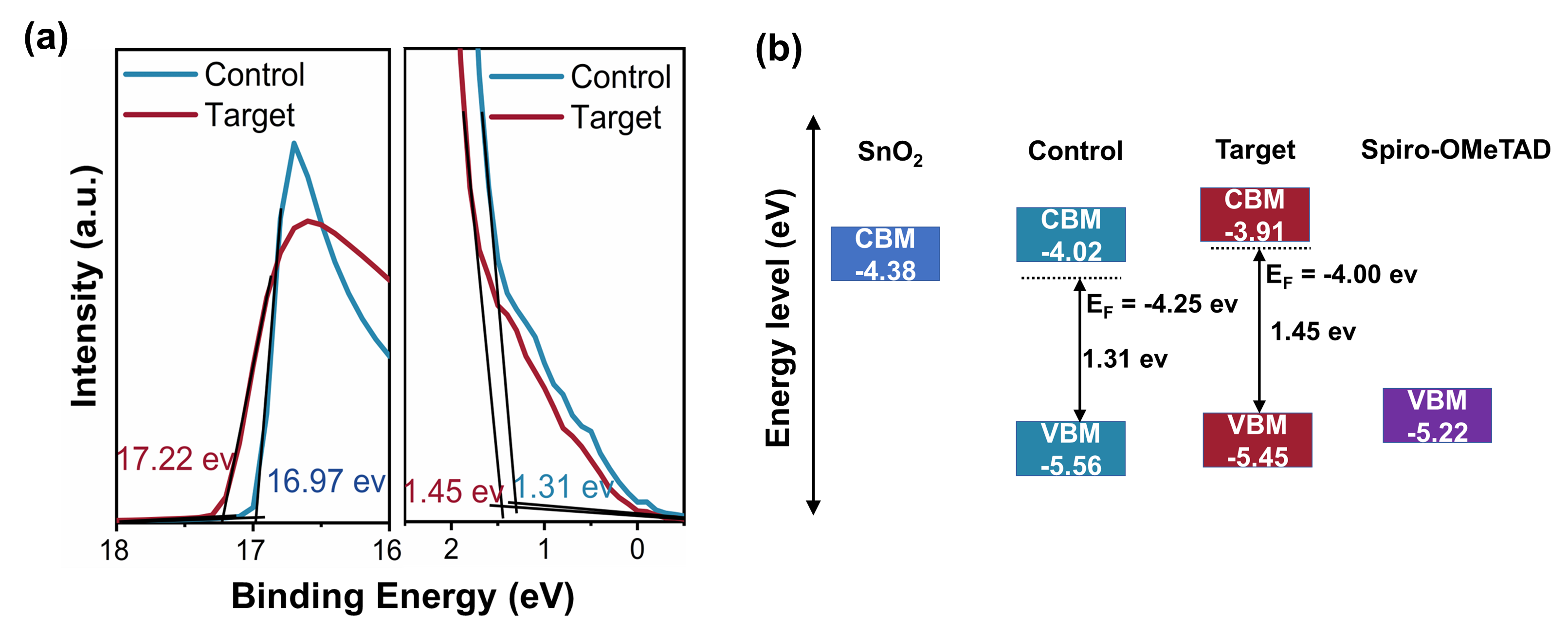


**Figure S23.** (a) Valence-band region and photoemission cut-off energy estimated from the UPS spectra. (b) Schematic of band energy levels of the control and target films.


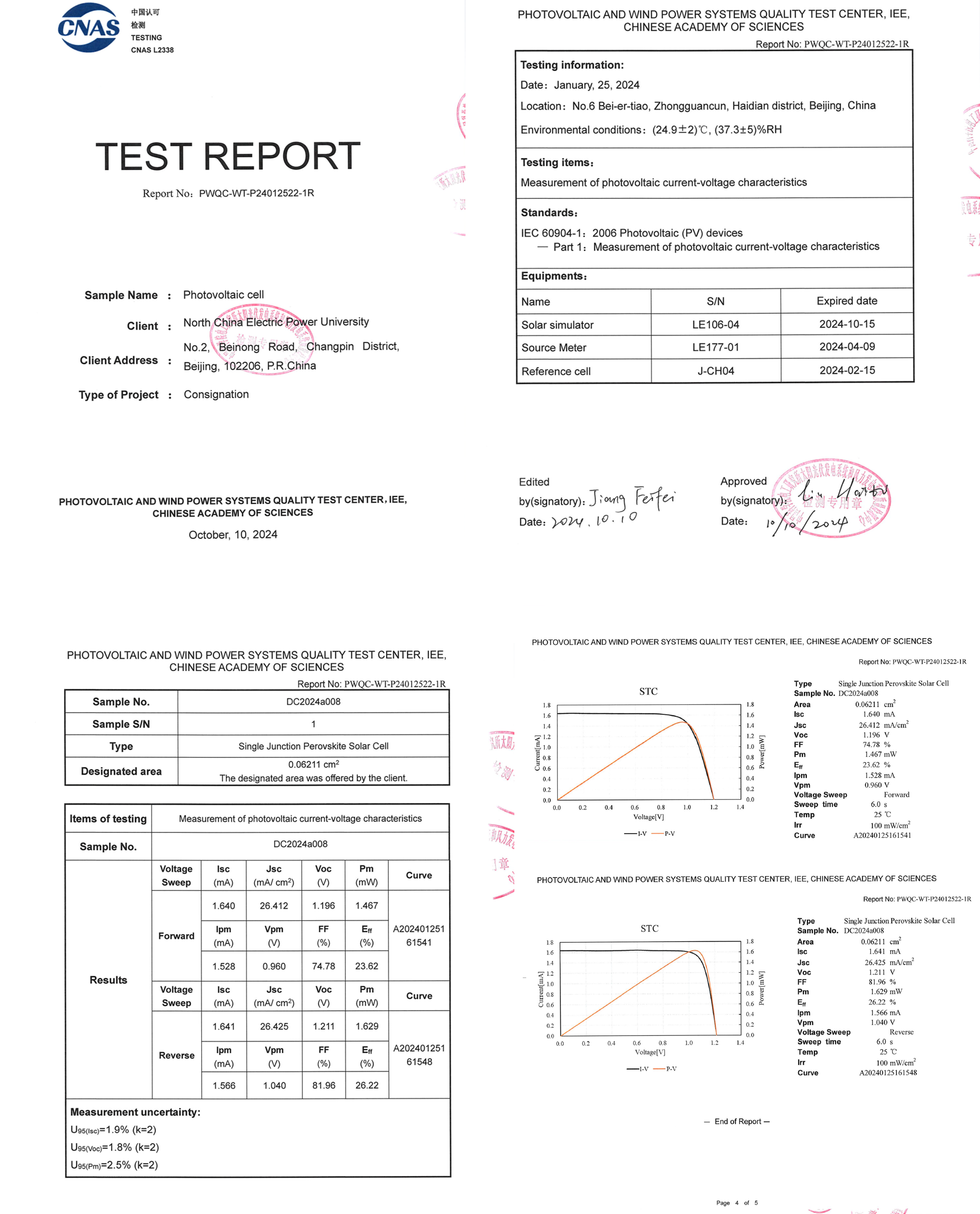


**Figure S24.** Certification reports of target device from Photovoltaic and Wind Power Systems Quality Test Center, IEE, Chinese Academy of Sciences.


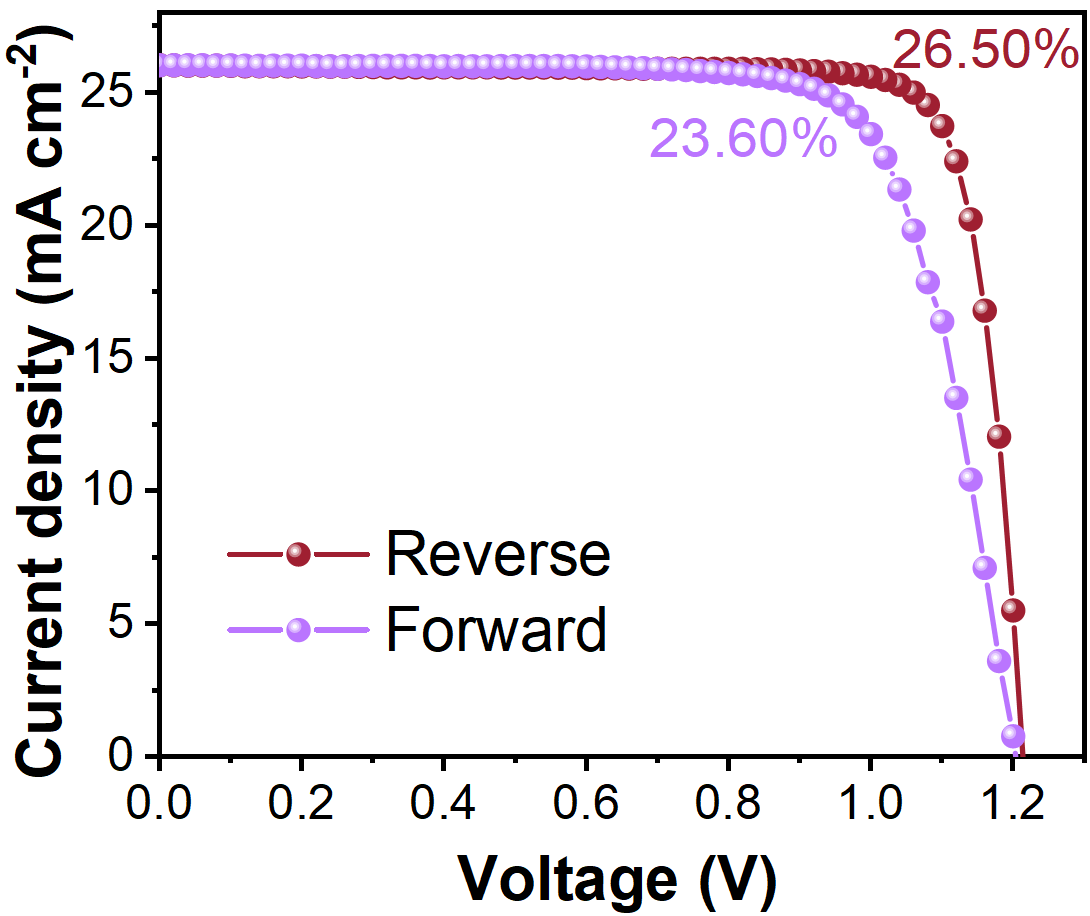


**Figure S25.** *J*−*V* plots of the champion target device measured in both forward and reverse scan directions.


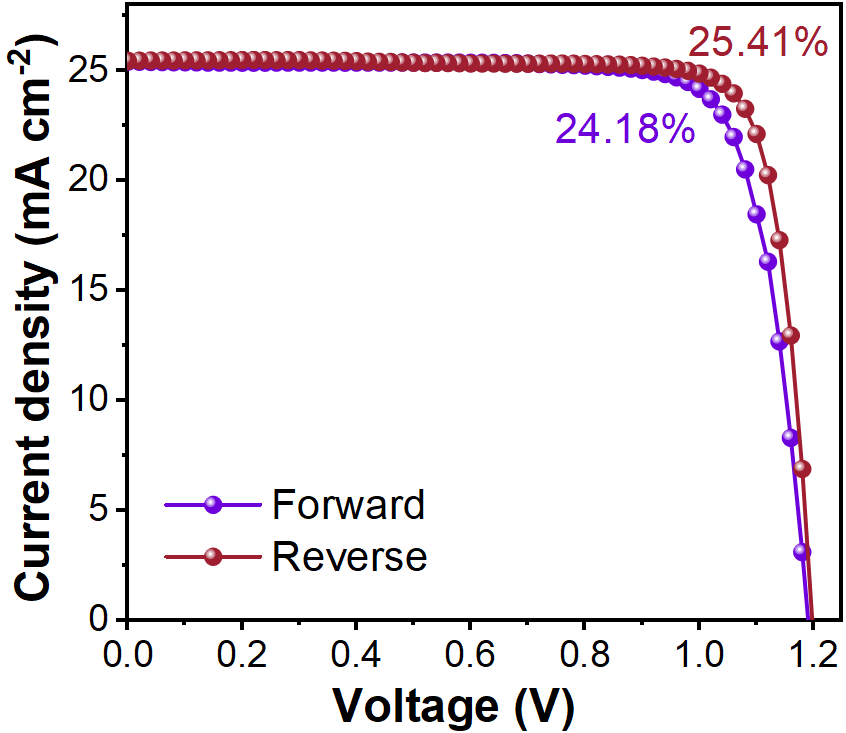


**Figure S26**. *J*−*V* plots of the target device fabricated under high-humidity conditions (~60% RH).


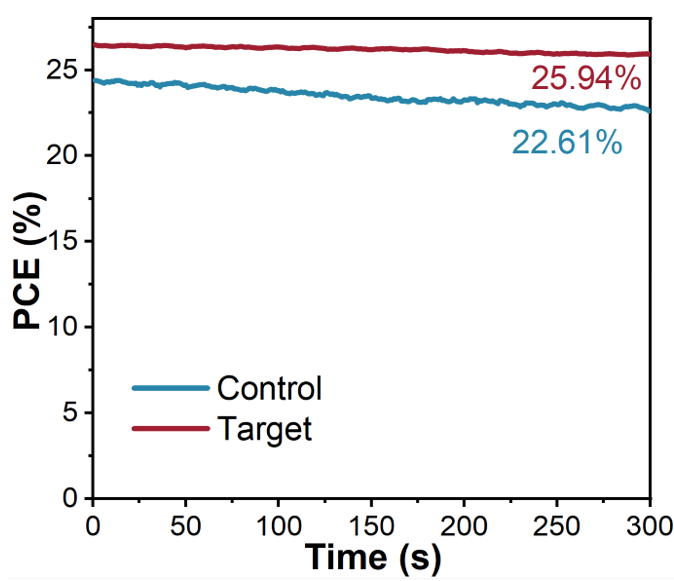


**Figure S27.** Stabilized efficiency of the control and target devices during maximum power point tracking (MPPT) over 300 seconds.


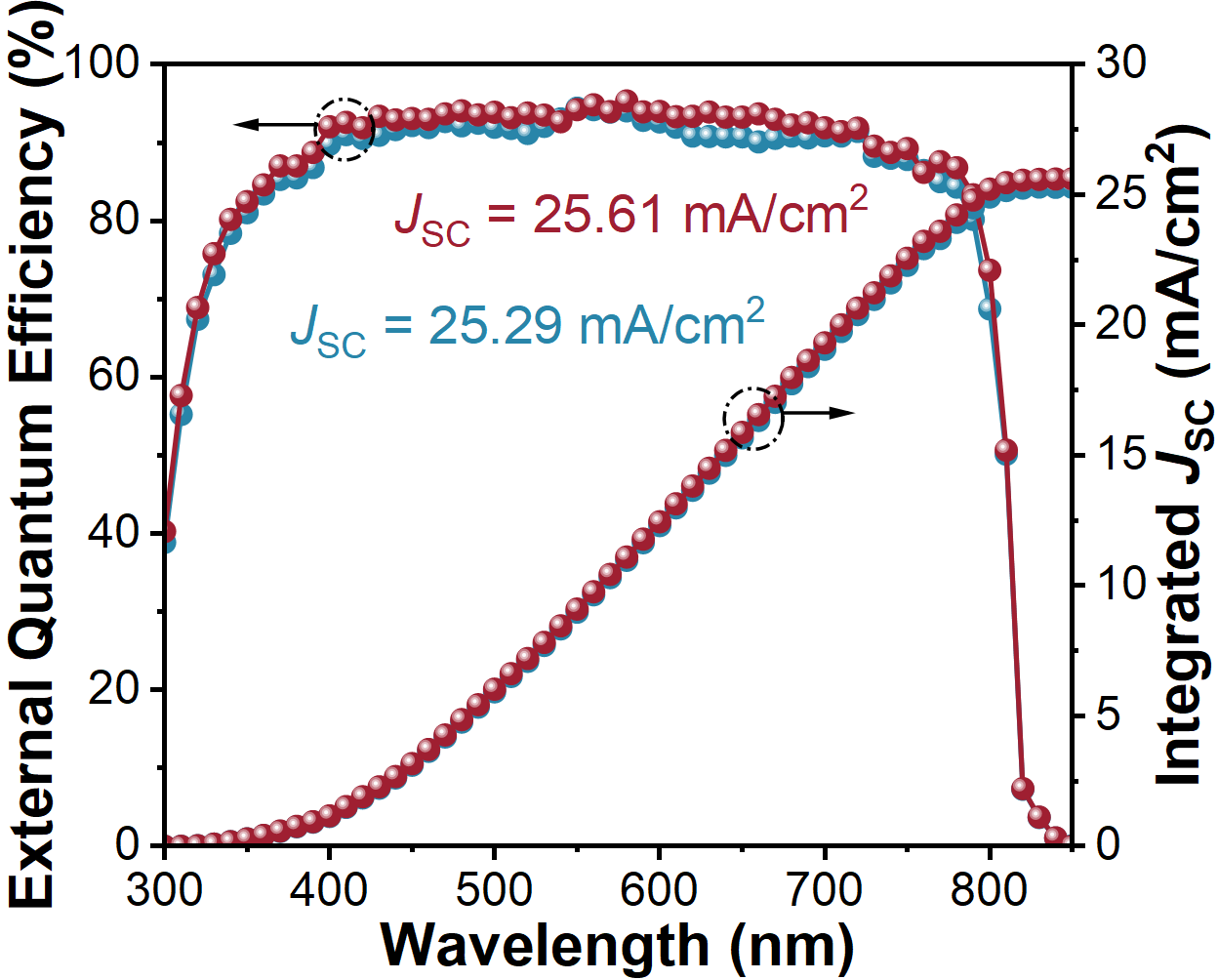


**Figure S28.** External quantum efficiency (EQE) spectra and integrated *J*sc for the champion control and target devices.


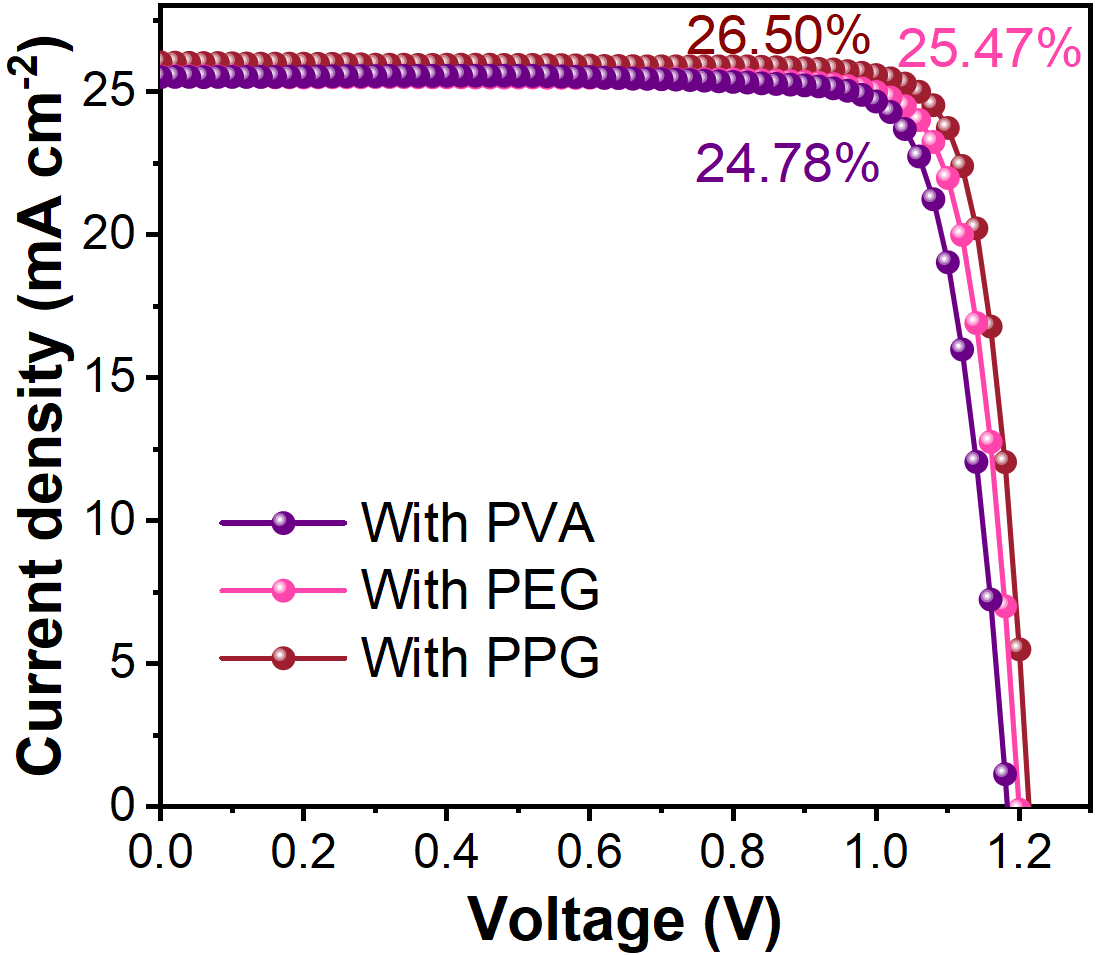


**Figure S29**. *J*−*V* plots of the PSCs fabricated using PVA-, PEG-, and PPG-based α-FAPbI_3_ microcrystals as precursors.


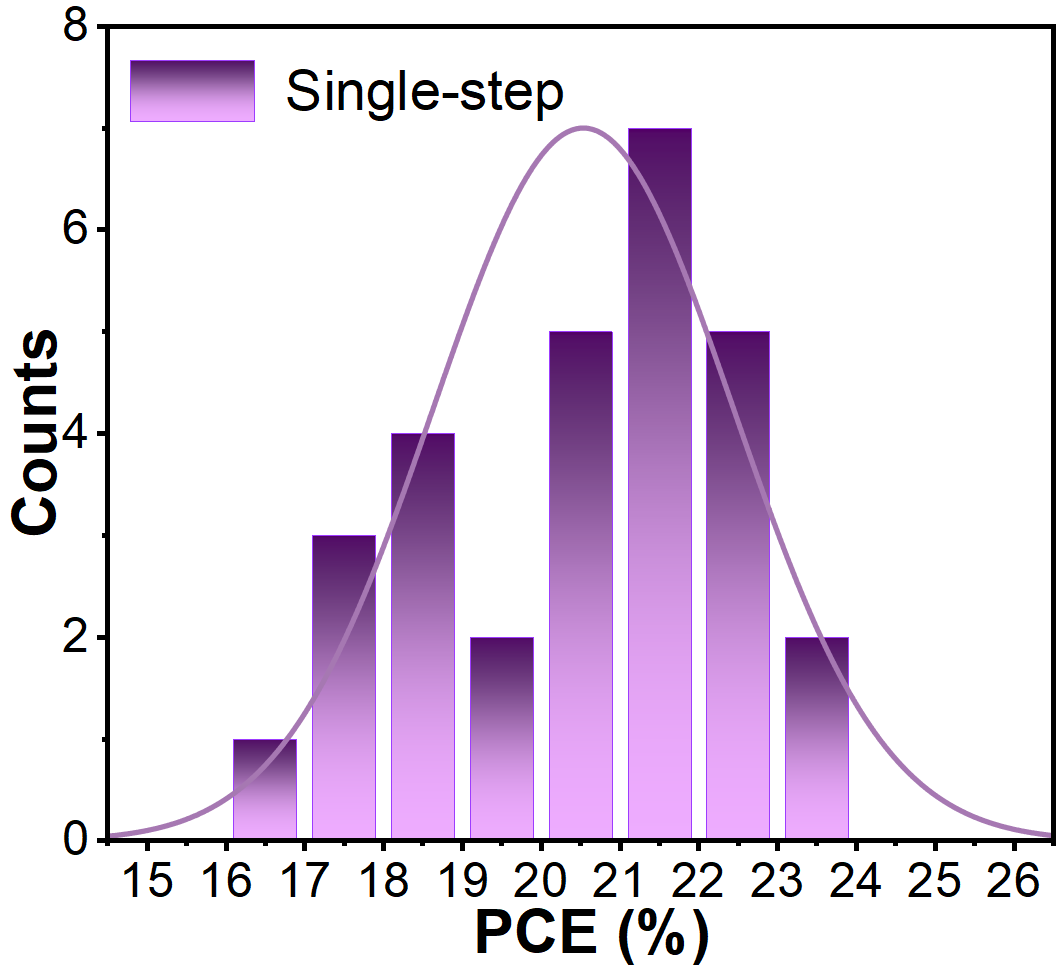


**Figure S30.** Power conversion efficiency (PCE) distribution of devices fabricated using single-step precursor route.


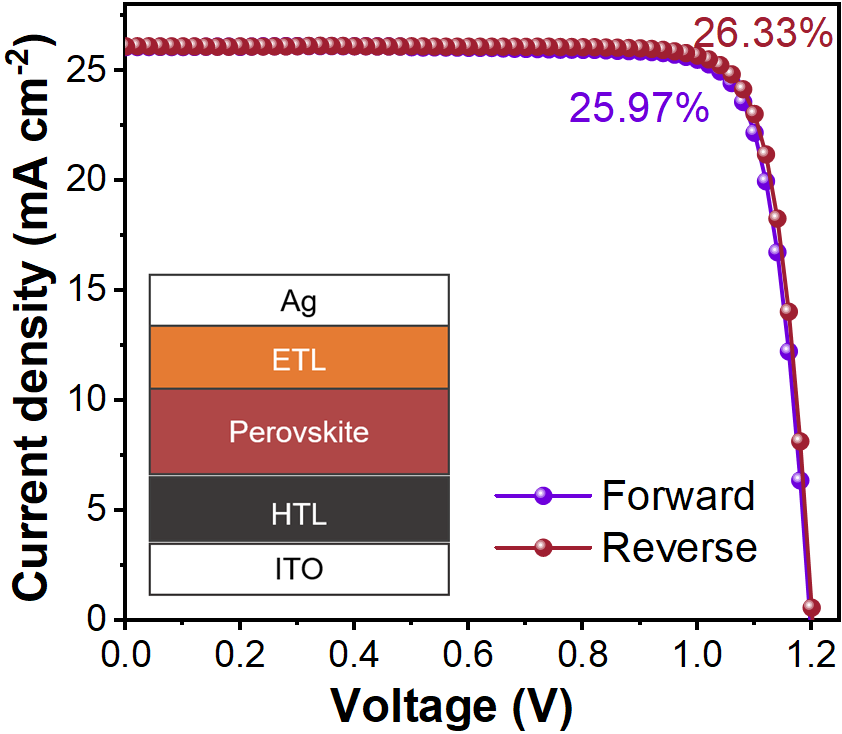


**Figure S31.** *J*−*V* characteristics of the champion p–i–n target device measured under both forward and reverse scan directions.


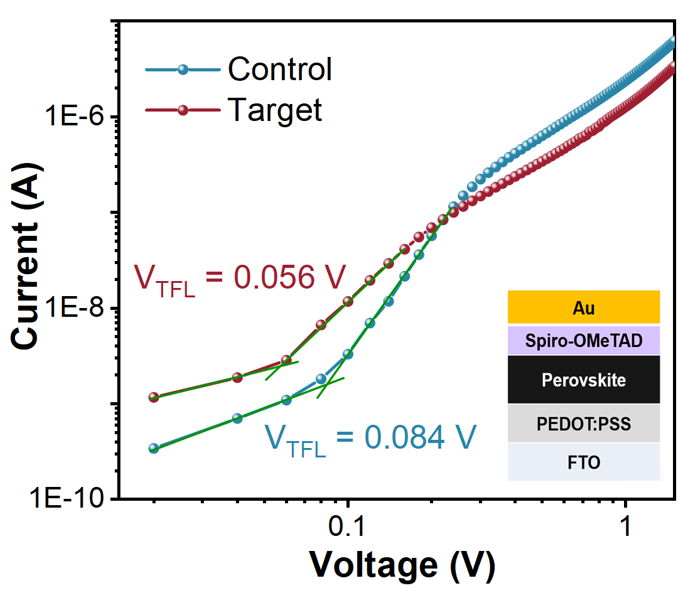


**Figure S32.** Space-charge-limited current (SCLC) curves of the hole-only (FTO/PEDOT/perovskite/spiro-OMeTAD/Au) devices.


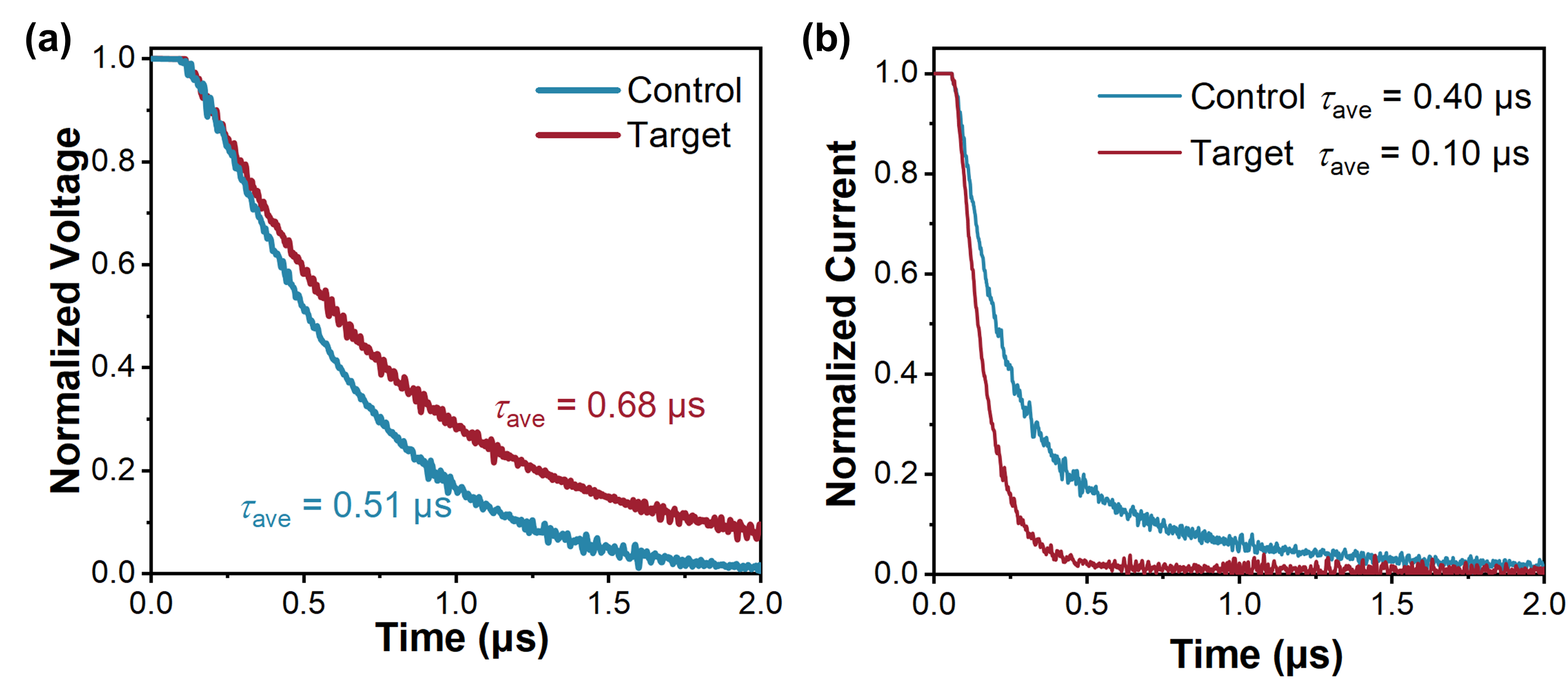


**Figure S33.** (a) Transient photovoltage (TPV) and (b) transient photocurrent (TPC) decay curves of the control and target devices.


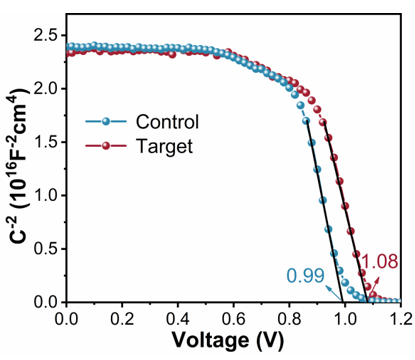


**Figure S34.** Mott-Schottky fitting of the capacitance-voltage (C^-2^-V) plots.


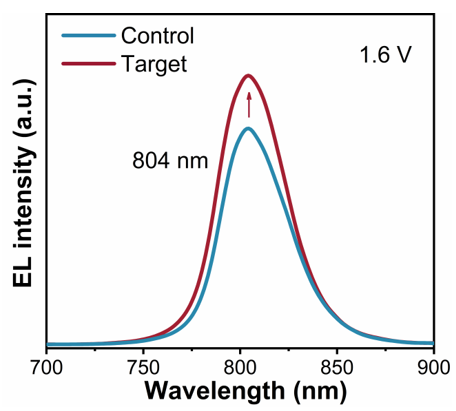


**Figure S35.** Electroluminescence spectra of control and target devices at 1.6 V bias.


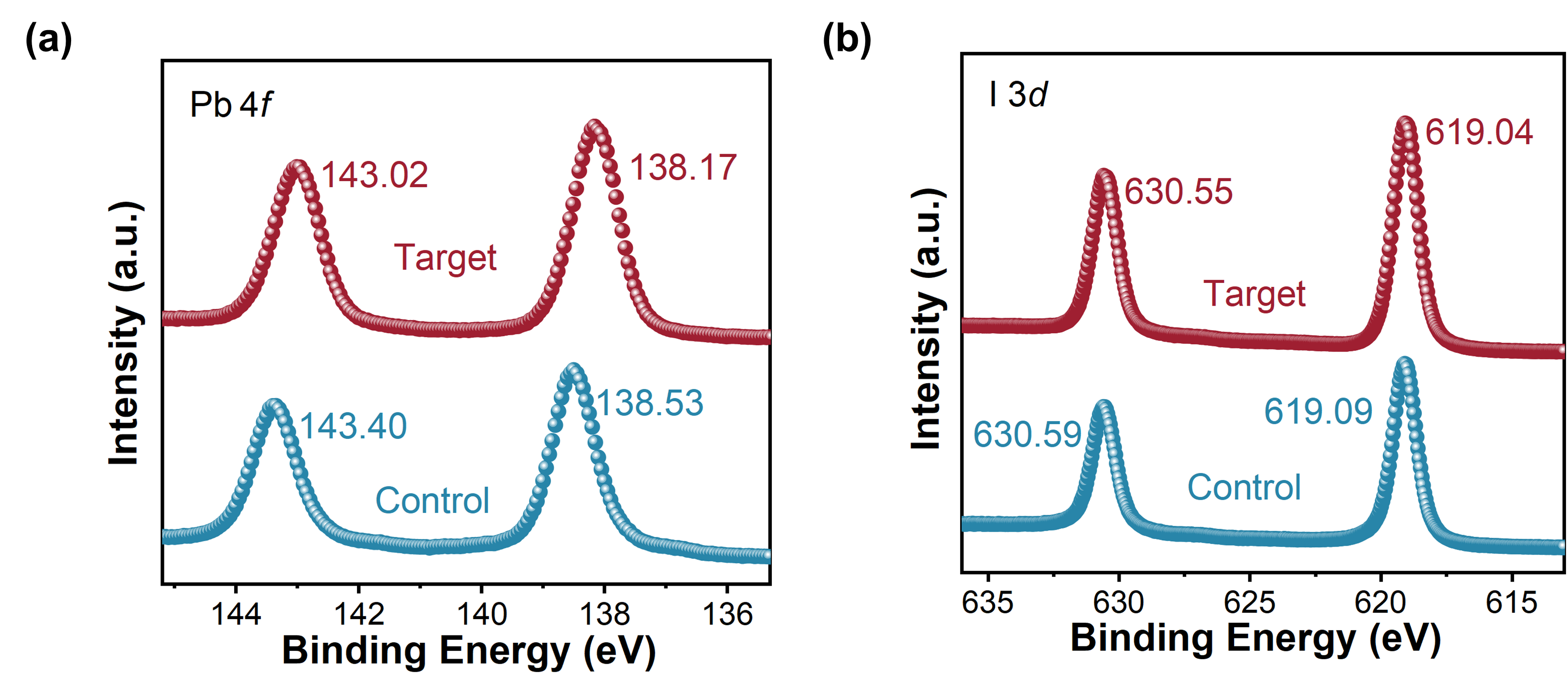


**Figure S36.** XPS spectra of (a) Pb 4*f* and (b) I 3*d* for the control and target films after thermal aging at 160 °C for 30 hours.


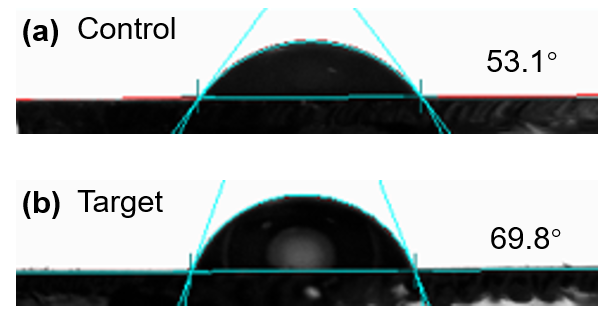


**Figure S37.** Water contact angles of the (a) control and (b) target films.


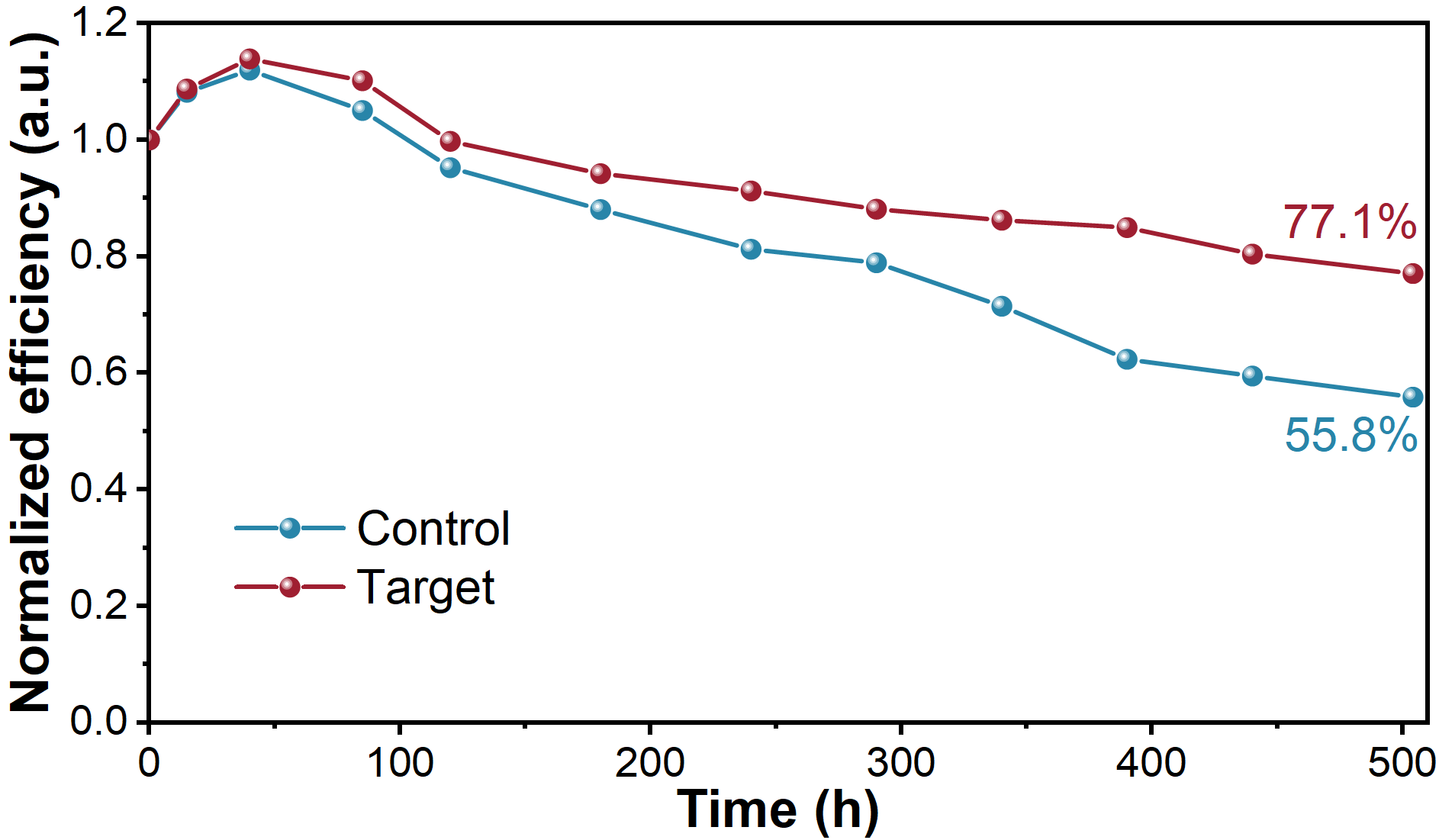


**Figure S38.** Thermal stability test of unencapsulated devices conducted at 85 °C in a nitrogen environment.


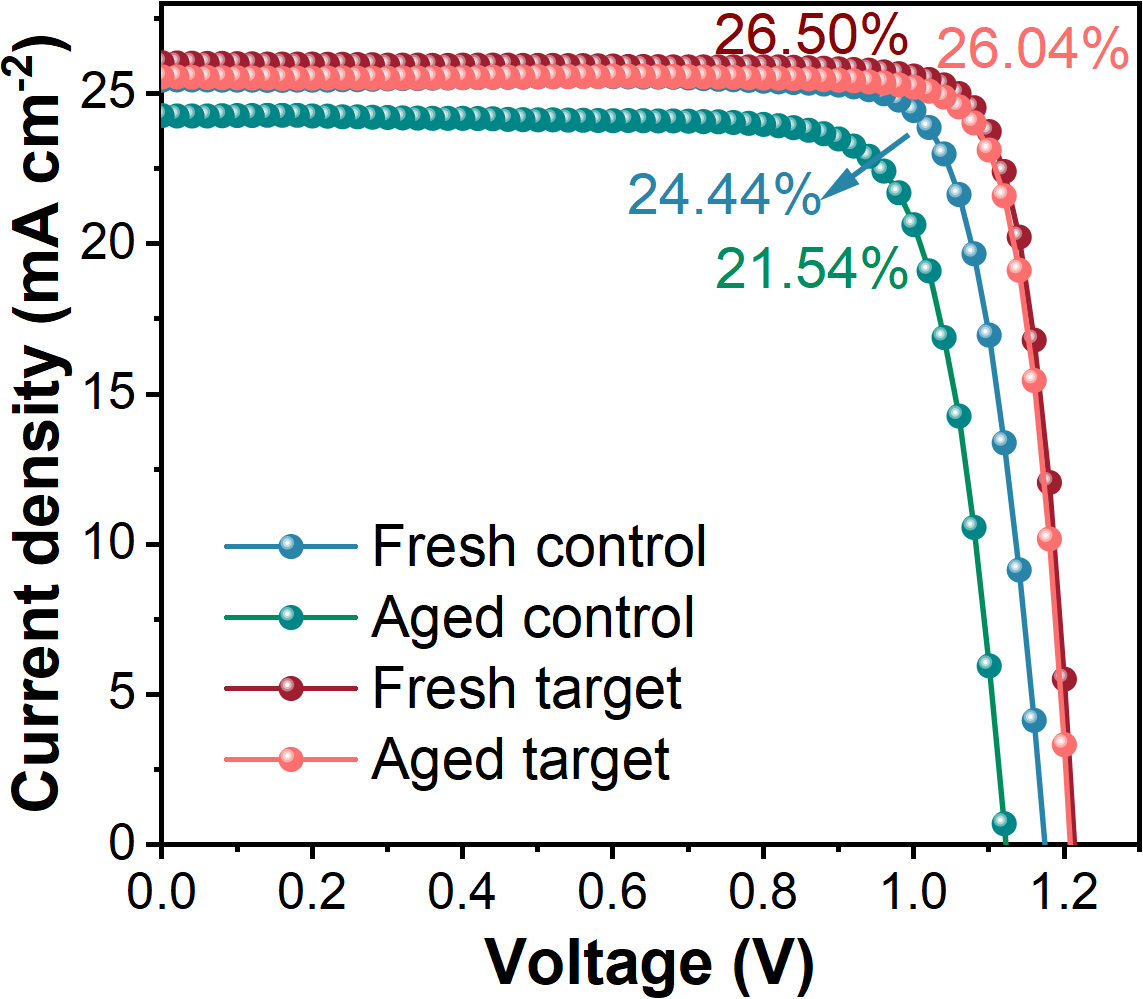


**Figure S39.** *J*-*V* curves of PSCs fabricated with fresh and aged perovskite microcrystals.


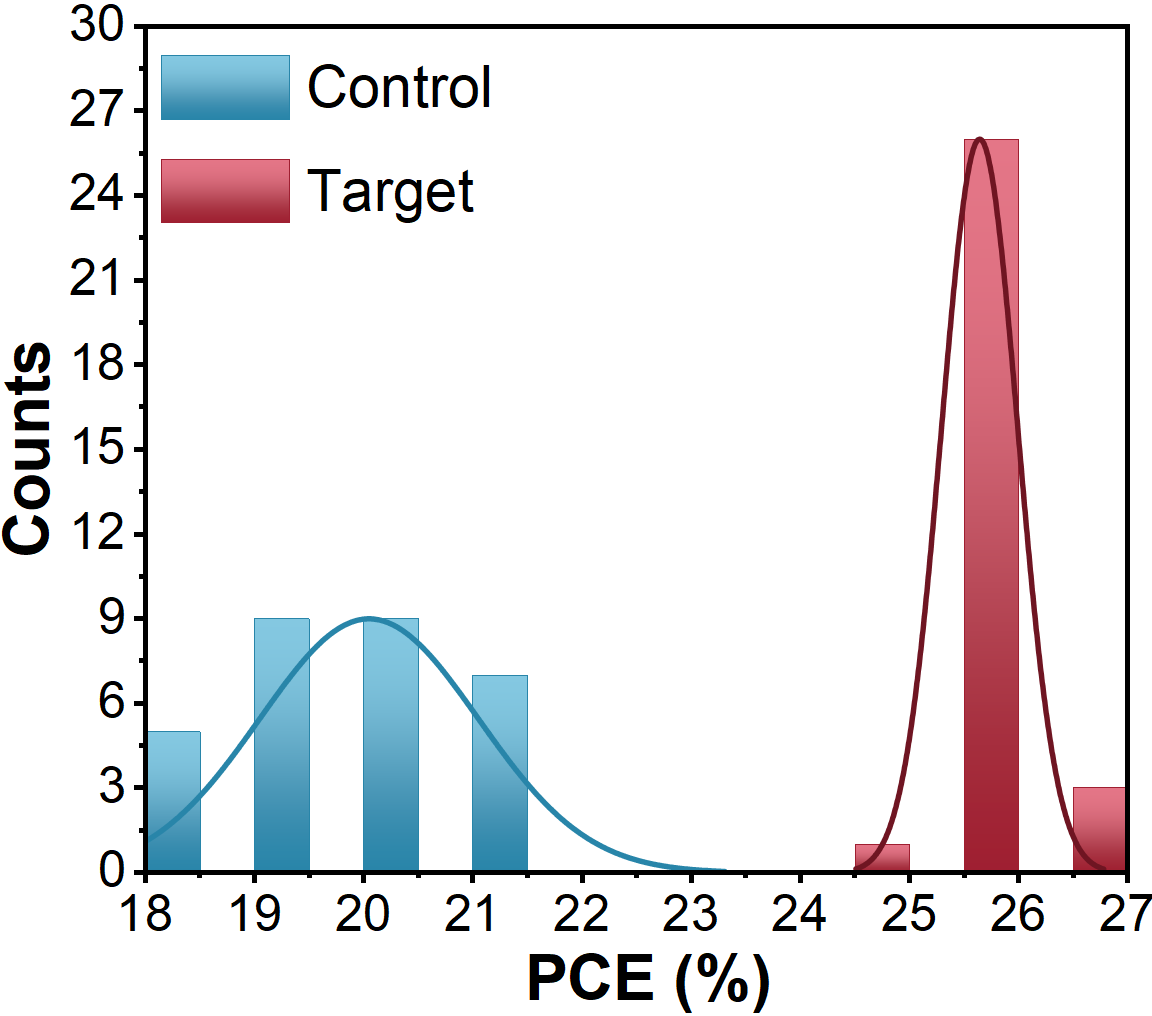


**Figure S40.** PCE distribution of devices fabricated using aged control and target microcrystals.

**Table S1.** Summary of materials used in fabrication of PSCs with PCEs exceeding 26%, as reported in recent literatures.

| Materials  ^Ref^ | Device structure | *J*_SC_ (mA/cm^2^) | Voc (V) | FF  (%) | PCE (%) | Certified  PCE (%) |
| --- | --- | --- | --- | --- | --- | --- |
| PbI_2_ (99.999%),  FAI (99.5%),  CsI (99.99%)  ^14^ | FTO/NiOx/Me-4PACz- 2DPAA/FA_0.95_Cs_0.05_PbI_3_  /PDAI_2_/C_60_/BCP/Ag | 25.95 | 1.20 | 85.43 | 26.53 | 26.02 |
| PbI_2_ (99.999%),  FAI (99.9%),  MACl (99.9%)  ^15^ | ITO/SnO_2_/Cs_0.05_FA_0.95_PbI_3_  /Spiro-OMeTAD/Ag | 26.12 | 1.19 | 83.85 | 26.15 | 25.59 |
| PbI_2_ (99.99%),  FAI (＞99.5%),  MACl  ^16^ | ITO/SnO_2_/FAPbI_3_-MASCN  /Spiro-OMeTAD/Au | 26.31 | 1.17 | 84.50 | 26.05 | 25.67 |
| α-phase FAPbI_3_ powder, MACl  ^17^ | FTO/TiO_2_-Li_2_CO_3_-BAE/  FAPbI_3_/Spiro-OMeTAD/Au | 26.28 | 1.18 | 85.90 | 26.52 | 26.31 |
| PbI_2_ (99.99%),  FAI (99.99%),  GAI (97%),  MAI (98%),  MACl (99.5%)  ^18^ | FTO/SnO_2_/FAPbI_3_/D18/Spiro-OMeTAD/Au | 26.54 | 1.19 | 83.92 | 26.39 | 26.17 |
| PbI_2_ (99.99%),  FAI (99.99%), MACl (99.5%)  ^19^ | FTO/SnO_2_-Nd_2_Cl_3_/FAPbI_3_/Spiro-OMeTAD/Au | 25.83 | 1.20 | 84.60 | 26.22 | / |
| PbI_2_ (＞99.99%),  FAI (＞99.5%),  MAI (＞99.5%),  CsI (＞99.99%),  MACl (＞99.5%)  ^20^ | FTO/Me-4PACz/Cs_0.05_(FA_0.98_MA_0.02_)_0.95_PbI_3_/PDI/C_60_/BCP/Cu | 25.67 | 1.18 | 86.47 | 26.15 | 25.87 |
| PbI_2_ (99.999%),  FAI, MACl  ^21^ | ITO/PTAA/MoS_2_/  FAPbI_3_/MoS_2_/C_60_/BCP/Ag | 25.76 | 1.20 | 85.62 | 26.37 | 26.21 |
| FAI (99.5%),  MAI (99.5%),  CsI (99.99%),  PbI_2_  ^22^ | FTO/Ph-4PACz/perovskite/C_60_/BCP/Ag | 25.70 | 1.20 | 85.39 | 26.38 | 25.87 |
| PbI_2_ (99.999%),  FAI (＞99.5%),  CsI (99.99%)  ^23^ | FTO/SnO_2_-CNCB/FA_0.9_Cs_0.1_PbI_3_/Spiro-OMeTAD/Au | 25.91 | 1.21 | 84.25 | 26.47 | / |
| PbI_2_ (99.999%),  FAI (＞99.5%),  MABr (＞99.5%),  CsI (＞99.9%),  MACl (＞99.5%)  ^24^ | ITO/SnO_2_/ Perovskite/Spiro-OMeTAD/Au | 25.97 | 1.19 | 84.21 | 26.04 | / |
| PbI_2_ (99.999%),  FAI,  MAI (99.9%),  CsI (99.98%),  MACl (99.9%)  ^25^ | FTO/NiOx/Me-4PACz/ Perovskite/C_60_/BCP/Ag | 26.17 | 1.19 | 84.24 | 26.17 | / |
| PbI_2_ (99.99%),  FAI (99.99%),  MAI (99.5%),  CsI (99.999%),  MACl (99.5%)  ^26^ | ITO/2PACz/HSCs/Perovskite/C_60_/BCP/Ag | 26.27 | 1.19 | 83.84 | 26.08 | 26.08 |
| FAI (99.99%),  MACl (99.99%),  PbI_2_ (≥99.99%),  PbBr_2_ (≥99.99%),  MABr (99.9%)  ^27^ | ITO/4PADCB/ Cs_0.05_(FA_0.98_MA_0.02_)_0.95_Pb(I_0.98_Br_0.02_)_3_ with BT2F-2B/C_60_ and BCP/Ag | 25.63 | 1.18 | 85.88 | 26.01 | / |
| MACl (99.9%),  PbI_2_ (99.999%),  FAI (99.99%),  CsI  ^28^ | ITO/SAM/ FA_0.95_Cs_0.05_PbI_3_/  PEABr/PCBM/BCP/Ag | 26.43 | 1.19 | 81.98 | 26.01 | / |
| FAI (99.9%),  MACl (99.9%),  MAI (99.9%),  PbI_2_ (99.999%),  ^29^ | ITO/Me-4PACz/Cs_0.05_FA_0.9_MA_0.05_PbI_3_/  ETM/BCP/Ag | 25.56 | 1.18 | 86.44 | 26.0 | 25.4 |
| PbI_2_ (99.99%),  PbBr_2_ (98%),  FAI (99.99%),  MAI (99.99%),  MABr (99.9%),  CsI (99.99%)  ^30^ | ITO/2PACz/FA_0.85_MA_0.10_Cs_0.05_Pb(I_0.98_Br_0.02_)_3_/2D-MAP/C_60_/BCP/Ag | 25.53 | 1.20 | 85.5 | 26.05 | 25.44 |
| PbI_2_ (99.999%),  FAI, CsI, MACl  ^31^ | FTO/TiO_2_/ FA_0.85_MA_0.10_Cs_0.05_PbI_3_/MeO-PEAI/Spiro-OMeTAD/Au | 26.21 | 1.19 | 83.44 | 26.0 | 25.4 |
| PbI_2_ (99.99%),  FAI (≥99.5%),  MACl (≥99.5%),  ^32^ | ITO/MPA-CPA/ Cs_0.05_MA_0.05_ FA_0.90_PbI_3_/Nd@C_82_/C_60_/BCP/Ag. | 26.26 | 1.19 | 85.97 | 26.78 | 26.29 |
| PbI_2_ (99.999%),  CsI (99.99%),  PbBr_2_ (99.999%),  FAI, MAI, MACl, MABr  ^33^ | FTO/NiOx/MeO-4PACz/ Cs_0.05_MA_0.05_ FA_0.90_PbI_3_/C_60_/BCP/Ag | 26.13 | 1.19 | 84.45 | 26.28 | / |
| PbI_2_ (99.999%),  CsI (99.99%),  PbBr_2_ (99.999%),  FAI, MAI, MACl, MABr  ^34^ | ITO/NiOx/MeO-2PACz/ Cs_0.05_FA_0.95_PbI_3_/  PEAI/PCBM/BCP/Ag | 26.36 | 1.19 | 85.27 | 26.83 | 26.32 |
| FAPbI_3_, MAPbI_3_, CsPbI_3_ microcrystals  ^35^ | FTO/SPCF-MeTPA/ Cs_0.05_FA_0.85_MA_0.1_PbI_3_/  C_60_/BCP/Cu | 25.76 | 1.19 | 86.3 | 26.35 | 25.75 |
| FAI (＞99.5%),  CsI (＞99.99%),  MABr (＞99.5%),  PbBr (＞99.99%),  MACl (＞99.5%)  ^36^ | FTO/4PABCz/Cs_0.05_(FA_0.98_MA_0.02_)_0.95_Pb(I_0.98_Br_0.02_)_3_/PDI/PCBM/BCP/Ag | 26.20 | 1.19 | 86.14 | 26.90 | 26.81 |
| PbI_2_ (99.99%),  FAI (＞99.99%),  CsI (99.99%),  MAI (99.5%),  MACl (99.5%)  ^37^ | ITO/HTM/SFT-STPA /Perovskite/C_60_/BCP/Cu | 25.72 | 1.18 | 86.1 | 26.1 | 25.7 |
| PbI_2_ (99.99%),  CsI (99.999%),  FAI, MACl  ^38^ | FTO/SnO_2_/Perovskite/ThPyI/Spiro-OMeTAD/Au | 26.09 | 1.21 | 83.13 | 26.16 | 25.84 |
| PbI_2_ (99.99%),  GAI (97%),  FAI (99.99%),  MAI (98%),  MACl (99.5%)  ^39^ | FTO/SnO_2_/Perovskite-METEAM/Spiro-OMeTAD/Au | 26.54 | 1.19 | 83.92 | 26.39 | 26.17 |
| PbI_2_ (99.999%),  FAI (99.8%),  MACl (99.99%)  ^40^ | FTO/SnO_2_/Perovskite/Spiro-OMeTAD/Au | 26.22 | 1.19 | 83.71 | 26.05 | 25.66 |
| black-phase FAPbI_3_, MACl  ^41^ | FTO/SnO_2_/Perovskite/Spiro-OMeTAD/Au | 25.94 | 1.18 | 85.27 | 26.05 | 25.54 |
| PbI_2_ (99.99%),  MACl (99%),  FAI, MAI, CsI  ^42^ | ITO/4PADCB/Perovskite/  C_60_/BCP/Cu | 25.84 | 1.18 | 85.8 | 26.27 | 25.94 |
| PbI_2_ (99.99%),  CsI (99.99%),  FAI, MAI, MACl, PbCl_2_  ^43^ | FTO/SAM/Perovskite/  C_60_/SnO_2_/Ag | 26.13 | 1.17 | 85.2 | 26.15 | 26.15 |
|  |  |  |  |  |  |  |

**Table S2.** Fitting results of TRPL spectra of the control and target perovskite films on glass substrates.

| Sample | *τ*_1_  (ns) | *τ*_2_  (ns) | A_1_ | A_2_ | *τ*_ave_  (ns) |
| --- | --- | --- | --- | --- | --- |
| Control | 1648.37 | 4040.24 | 143.20 | 321.03 | 3671.98 |
| Target | 899.48 | 5402.03 | 43.79 | 401.99 | 5321.82 |

**Table S3.** Key photovoltaic parameters of the champion control and target devices, measured from reverse scan (1.3 to –0.2 V) and forward scan (–0.2 to 1.3 V) under simulated AM1.5 G solar irradiation with an intensity of 100 mW cm^-2^.

| Champion device | Scan direction | *V*_OC_  (V) | *J*_SC_  (mA cm^-2^) | *FF*  (%) | PCE  (%) |
| --- | --- | --- | --- | --- | --- |
| Control | Reverse | 1.174 | 25.45 | 81.77 | 24.44 |
|  | Forward | 1.147 | 25.50 | 72.03 | 21.07 |
| Target | Reverse | 1.214 | 26.03 | 83.90 | 26.50 |
|  | Forward | 1.204 | 26.03 | 75.34 | 23.60 |

**Table S4**. Photovoltaic parameters of the target device fabricated under high-humidity conditions (~60% RH), measured under reverse and forward scan directions under simulated AM1.5G illumination (100 mW cm^-2^).

| Device | Scan direction | *V*_OC_  (V) | *J*_SC_  (mA cm^-2^) | *FF*  (%) | PCE  (%) |
| --- | --- | --- | --- | --- | --- |
| Target | Reverse | 1.197 | 25.46 | 83.37 | 25.41 |
|  | Forward | 1.190 | 25.42 | 79.93 | 24.18 |

**Table S5.** Photovoltaic parameters of the champion target perovskite solar module.

| *V*oc  (V) | *I*sc  (mA) | *FF*  (%) | PCE  (%) |
| --- | --- | --- | --- |
| 9.27 | 89.5 | 79.2 | 22.66 |

**Table S6.** Photovoltaic parameters of PSCs fabricated using PVA-, PEG-, and PPG-based α-FAPbI_3_ microcrystals as precursors.

| Devices | *V*oc  (V) | *J*sc  (mA cm^-2^) | *FF*  (%) | PCE  (%) |
| --- | --- | --- | --- | --- |
| With PVA | 1.183 | 25.52 | 82.07 | 24.78 |
| With PEG | 1.200 | 25.54 | 83.14 | 25.47 |
| With PPG | 1.214 | 26.03 | 83.90 | 26.50 |

**Table S7**. Photovoltaic parameters of champion the device fabricated using the perovskite films obtained from the single-step precursor route (FAI, PbI_2_, MACl, CsCl and PPG in DMF/DMSO).

| Scan direction | *V*_OC_  (V) | *J*_SC_  (mA cm^-2^) | *FF*  (%) | PCE  (%) |
| --- | --- | --- | --- | --- |
| Reverse | 1.163 | 25.43 | 79.33 | 23.46 |
| Forward | 1.166 | 25.42 | 65.15 | 19.30 |

**Table S8.** Photovoltaic parameters of the champion p–i–n target device, measured under reverse and forward scan directions under simulated AM1.5G illumination (100 mW cm^-2^).

| p-i-n  device | Scan direction | *V*_OC_  (V) | *J*_SC_  (mA cm^-2^) | *FF*  (%) | PCE  (%) |
| --- | --- | --- | --- | --- | --- |
| Target | Reverse | 1.201 | 26.11 | 83.94 | 26.33 |
|  | Forward | 1.197 | 26.08 | 83.16 | 25.97 |

**References**

(1) Jeong, J.; Kim, M.; Seo, J.; Lu, H.; Ahlawat, P.; Mishra, A.; Yang, Y.; Hope, M. A.; Eickemeyer, F. T.; Kim, M. Pseudo-Halide Anion Engineering for α-FAPbI_3_ Perovskite Solar Cells. *Nature* **2021**, *592* (7854), 381-385.

(2) Peng, W.; Mao, K.; Cai, F.; Meng, H.; Zhu, Z.; Li, T.; Yuan, S.; Xu, Z.; Feng, X.; Xu, J. Reducing nonradiative recombination in perovskite solar cells with a porous insulator contact. *Science* **2023**, *379* (6633), 683-690.

(3) Fan, Y.; Qin, Z.; Lu, L.; Zhang, N.; Liang, Y.; Wang, S.; Zhan, W.; Guo, J.; Wang, H.; Chen, Y. An efficient and precise solution-vacuum hybrid batch fabrication of 2D/3D perovskite submodules. *Nature Commun.* **2025**, *16* (1), 7019.

(4) Bochun, K.; Yan, F. Emerging strategies for the large-scale fabrication of perovskite solar modules: from design to process. *Energy Environ. Sci.* **2025**, *18* (9), 3917-3954.

(5) Yang, Y.; Cheng, S.; Zhu, X.; Li, S.; Zheng, Z.; Zhao, K.; Ji, L.; Li, R.; Liu, Y.; Liu, C. Inverted Perovskite Solar Cells with over 2,000 h Operational Stability at 85° C Using Fixed Charge Passivation. *Nat. Energy* **2024**, *9* (1), 37-46.

(6) Duijnstee, E. A.; Gallant, B. M.; Holzhey, P.; Kubicki, D. J.; Collavini, S.; Sturdza, B. K.; Sansom, H. C.; Smith, J.; Gutmann, M. J.; Saha, S. Understanding the Degradation of Methylenediammonium and its Role in Phase-Stabilizing Formamidinium Lead Triiodide. *J. Am. Chem. Soc.* **2023**, *145* (18), 10275-10284.

(7) Lu, H.; Liu, Y.; Ahlawat, P.; Mishra, A.; Tress, W. R.; Eickemeyer, F. T.; Yang, Y.; Fu, F.; Wang, Z.; Avalos, C. E. Vapor-Assisted Deposition of Highly Efficient, Stable Black-Phase FAPbI_3_ Perovskite Solar Cells. *Science* **2020**, *370* (6512), eabb8985.

(8) Kresse, G.; Furthmüller, J. Efficient Iterative Schemes for ab Initio Total-energy Calculations Using a Plane-Wave Basis Set. *Phys. Rev. B* **1996**, *54* (16), 11169.

(9) Perdew, J. P.; Burke, K.; Ernzerhof, M. Generalized Gradient Approximation Made Simple. *Phys. Rev. Lett.* **1996**, *77* (18), 3865.

(10) Kresse, G.; Joubert, D. From Ultrasoft Pseudopotentials to the Projector Augmented-wave Method. *Phys. Rev. B* **1999**, *59* (3), 1758.

(11) Grimme, S.; Antony, J.; Ehrlich, S.; Krieg, H. A Consistent and Accurate ab initio Parametrization of Density Functional Dispersion Correction (DFT-D) for the 94 Elements H-Pu. *J. Chem. Phys.* **2010**, *132* (15).

(12) Grimme, S.; Ehrlich, S.; Goerigk, L. Effect of the Damping Function in Dispersion Corrected Density Functional Theory. *J. Comput. Chem.* **2011**, *32* (7), 1456-1465.

(13) Monkhorst, H. J.; Pack, J. D. Special Points for Brillouin-zone Integrations. *Phys. Rev. B* **1976**, *13* (12), 5188.

(14) Peng, Y.; Chen, Y.; Zhou, J.; Luo, C.; Tang, W.; Duan, Y.; Wu, Y.; Peng, Q. Enlarging moment and regulating orientation of buried interfacial dipole for efficient inverted perovskite solar cells. *Nature Commun.* **2025**, *16* (1), 1252.

(15) Shao, C.; Ma, J.; Niu, G.; Nie, Z.; Zhao, Y.; Wang, F.; Wang, J. Strain Release via Glass Transition Temperature Regulation for Efficient and Stable Perovskite Solar Cells. *Adv. Mater.* **2025**, 2417150.

(16) Li, M.; Xie, Y.; Luo, L.; Zheng, Z.; Guo, J.; He, L.; Zheng, X.; Liu, R.; Rong, Y.; Guo, R. In Situ Impurity Phase Repair Strategy Enables Highly‐Efficient Perovskite Solar Cells with Periodic Photovoltaic Performance. *Adv. Mater.* **2025**, 2501057.

(17) Li, Q.; Liu, H.; Hou, C.-H.; Yan, H.; Li, S.; Chen, P.; Xu, H.; Yu, W.-Y.; Zhao, Y.; Sui, Y. Harmonizing the bilateral bond strength of the interfacial molecule in perovskite solar cells. *Nat. Energy* **2024**, *9* (12), 1506-1516.

(18) Shen, L.; Song, P.; Jiang, K.; Zheng, L.; Qiu, J.; Li, F.; Huang, Y.; Yang, J.; Tian, C.; Jen, A. K.-Y. Ultrathin polymer membrane for improved hole extraction and ion blocking in perovskite solar cells. *Nat. Commun.* **2024**, *15* (1), 10908.

(19) Wan, Z.; Li, C.; Jia, C.; Su, J.; Li, Z.; Chen, Y.; Rao, F.; Cao, F.; Xue, J.; Shi, J. Suppressing Ion Migration through Dual Interface Engineering toward Efficient and Stable Perovskite Solar Modules. *ACS Energy Lett.* **2025**, *10* (4), 1585-1595.

(20) Zheng, Y.; Li, Y.; Zhuang, R.; Wu, X.; Tian, C.; Sun, A.; Chen, C.; Guo, Y.; Hua, Y.; Meng, K. Towards 26% efficiency in inverted perovskite solar cells via interfacial flipped band bending and suppressed deep-level traps. *Energy Environ. Sci.* **2024**, *17* (3), 1153-1162.

(21) Zai, H.; Yang, P.; Su, J.; Yin, R.; Fan, R.; Wu, Y.; Zhu, X.; Ma, Y.; Zhou, T.; Zhou, W. Wafer-scale monolayer MoS_2_ film integration for stable, efficient perovskite solar cells. *Science* **2025**, *387* (6730), 186-192.

(22) Zhu, Z.; Ke, B.; Sun, K.; Jin, C.; Song, Z.; Jiang, R.; Li, J.; Kong, S.; Liu, C.; Bai, S. High-performance inverted perovskite solar cells and modules via aminothiazole passivation. *Energy Environ. Sci.* **2025**.

(23) Li, Y.; Dong, L.; Cai, Y.; Li, Y.; Xu, D.; Lei, H.; Li, N.; Fan, Z.; Tan, J.; Sun, R. Meticulous Design of High‐Polarity Interface Material for FACsPbI_3_ Perovskite Solar Cells with Efficiency of 26.47%. *Angew. Chem. Int. Ed.*, e202504902.

(24) Cai, Z.; Yao, Z.; Xing, Z.; Dai, R.; Huang, Z.; Meng, X.; Hu, X.; Chen, Y. A Buried Interface Fastening Approach for Efficient and Flexible Perovskite Photovoltaics. *Adv. Funct. Mater.* **2025**, 2505921.

(25) Wang, M.; Li, L.; Wang, J.; Huang, H.; Cui, P.; Lan, Z.; Qu, S.; Suo, Y.; Li, M. Accelerating direct formation of α-FAPbl_3_ by dual-additives synergism for inverted perovskite solar cells with efficiency exceeding 26%. *Chem. Eng. J.* **2025**, *505*, 159056.

(26) Dong, B.; Wei, M.; Li, Y.; Yang, Y.; Ma, W.; Zhang, Y.; Ran, Y.; Cui, M.; Su, Z.; Fan, Q. Self-assembled bilayer for perovskite solar cells with improved tolerance against thermal stresses. *Nat. Energy* **2025**, 1-12.

(27) Song, Z.; Sun, K.; Meng, Y.; Zhu, Z.; Wang, Y.; Zhang, W.; Bai, Y.; Lu, X.; Tian, R.; Liu, C. Universal Approach for Managing Iodine Migration in Inverted Single‐Junction and Tandem Perovskite Solar Cells. *Adv. Mater.* **2025**, *37* (3), 2410779.

(28) Chen, C. H.; Liu, G. W.; Chen, X.; Deger, C.; Jin, R. J.; Wang, K. L.; Chen, J.; Xia, Y.; Huang, L.; Yavuz, I. Methylthio Substituent in SAM Constructing Regulatory Bridge with Photovoltaic Perovskites. *Angew. Chem. Int. Ed.* **2025**, *64* (7), e202419375.

(29) Feng, K.; Wang, G.; Lian, Q.; Gámez-Valenzuela, S.; Li, B.; Ding, R.; Yang, W.; Wang, K.; Zeng, J.; Zhang, Y. Non-fullerene electron-transporting materials for high-performance and stable perovskite solar cells. *Nat. Mater.* **2025**, 1-8.

(30) Chang, X.; Azmi, R.; Yang, T.; Wu, N.; Jeong, S. Y.; Xi, H.; Satrio Utomo, D.; Vishal, B.; Isikgor, F. H.; Faber, H. Solvent-dripping modulated 3D/2D heterostructures for high-performance perovskite solar cells. *Nature Commun.* **2025**, *16* (1), 1042.

(31) Qu, S.; Huang, H.; Wang, J.; Cui, P.; Li, Y.; Wang, M.; Li, L.; Yang, F.; Sun, C.; Zhang, Q. Revealing and Inhibiting the Facet‐related Ion Migration for Efficient and Stable Perovskite Solar Cells. *Angew. Chem.* **2025**, *137* (4), e202415949.

(32) Lin, Y.; Lin, Z.; Lv, S.; Shui, Y.; Zhu, W.; Zhang, Z.; Yang, W.; Zhao, J.; Gu, H.; Xia, J. A Nd@ C_82_-polymer interface for efficient and stable perovskite solar cells. *Nature* **2025**, 1-3.

(33) Zhang, Z.; Feng, Y.; Ding, J.; Ma, Q.; Zhang, H.; Zhang, J.; Li, M.; Geng, T.; Gao, W.; Wang, Y. Rationally designed universal passivator for high-performance single-junction and tandem perovskite solar cells. *Nature Commun.* **2025**, *16* (1), 753.

(34) Lu, M.; Ding, J.; Ma, Q.; Zhang, Z.; Li, M.; Gao, W.; Mo, W.; Zhang, B.; Pauporté, T.; Zhang, J. Dual-site passivation by heterocycle functionalized amidinium cations toward high-performance inverted perovskite solar cells and modules. *Energy Environ. Sci.* **2025**.

(35) Liu, Z.; Zeng, J.; Wang, D.; Zhu, P.; Wang, L.; Bao, Y.; Xu, Y.; Peng, W.; He, S.; Lei, Z. Spiro-Linked Planar Core Small Molecule Hole Transport Materials Enabling High-Performance Inverted Perovskite Solar Cells. *J. Am. Chem. Soc.* **2025**.

(36) Du, J.; Chen, J.; Ouyang, B.; Sun, A.; Tian, C.; Zhuang, R.; Chen, C.; Liu, S.; Chen, Q.; Li, Z. Face-on Oriented Self-Assembled Molecules with Enhanced π-π Stacking for Highly Efficient Inverted Perovskite Solar Cells on Rough FTO Substrate. *Energy Environ. Sci.* **2025**.

(37) Zeng, J.; Liu, Z.; Wang, D.; Wu, J.; Zhu, P.; Bao, Y.; Guo, X.; Qu, G.; Hu, B.; Wang, X. Small-molecule hole transport materials for> 26% efficient inverted perovskite solar cells. *J. Am. Chem. Soc.* **2024**, *147* (1), 725-733.

(38) Gao, Y.; Song, Z.; Fu, Q.; Chen, Y.; Yang, L.; Hu, Z.; Chen, Y.; Liu, Y. Controlled Nucleation and Oriented Crystallization of Methylammonium‐Free Perovskites via In‐Situ Generated 2D Perovskite Phases. *Adv. Mater.* **2024**, *36* (33), 2405921.

(39) Shen, L.; Song, P.; Jiang, K.; Zheng, L.; Qiu, J.; Li, F.; Huang, Y.; Yang, J.; Tian, C.; Jen, A. K.-Y. Ultrathin polymer membrane for improved hole extraction and ion blocking in perovskite solar cells. *Nature Commun.* **2024**, *15* (1), 10908.

(40) Zhang, Q.; Huang, H.; Yang, Y.; Wang, M.; Qu, S.; Lan, Z.; Jiang, T.; Wang, Z.; Du, S.; Lu, Y. A Universal Ternary Solvent System of Surface Passivator Enables Perovskite Solar Cells with Efficiency Exceeding 26%. *Adv. Mater.* **2024**, *36* (50), 2410390.

(41) Paik, M. J.; Kim, Y. Y.; Kim, J.; Park, J.; Seok, S. I. Ultrafine SnO_2_ colloids with enhanced interface quality for high-efficiency perovskite solar cells. *Joule* **2024**, *8* (7), 2073-2086.

(42) Wen, Y.; Zhang, T.; Wang, X.; Liu, T.; Wang, Y.; Zhang, R.; Kan, M.; Wan, L.; Ning, W.; Wang, Y. Amorphous (lysine) _2_PbI_2_ layer enhanced perovskite photovoltaics. *Nature Commun.* **2024**, *15* (1), 7085.

(43) Chen, H.; Liu, C.; Xu, J.; Maxwell, A.; Zhou, W.; Yang, Y.; Zhou, Q.; Bati, A. S.; Wan, H.; Wang, Z. Improved charge extraction in inverted perovskite solar cells with dual-site-binding ligands. *Science* **2024**, *384* (6692), 189-193.
